# spSeudoMap: cell type mapping of spatial transcriptomics using unmatched single-cell RNA-seq data

**DOI:** 10.1186/s13073-023-01168-5

**Published:** 2023-03-17

**Authors:** Sungwoo Bae, Hongyoon Choi, Dong Soo Lee

**Affiliations:** 1 Institute of Radiation Medicine, Medical Research Center, Seoul National University, Seoul, Republic of Korea; 2 Department of Nuclear Medicine, Seoul National University Hospital, 101, Daehak-ro, Jongno-gu, Seoul, 03080 Republic of Korea; 3 Department of Nuclear Medicine, Seoul National University College of Medicine, Seoul, Republic of Korea; 4 Portrai, Inc., Seoul, Republic of Korea

**Keywords:** Spatial transcriptomics, Single-cell RNA-seq, Cell sorting, Cell type mapping, Synthetic cell mixture, Pseudobulk

## Abstract

Since many single-cell RNA-seq (scRNA-seq) data are obtained after cell sorting, such as when investigating immune cells, tracking cellular landscape by integrating single-cell data with spatial transcriptomic data is limited due to cell type and cell composition mismatch between the two datasets. We developed a method, spSeudoMap, which utilizes sorted scRNA-seq data to create virtual cell mixtures that closely mimic the gene expression of spatial data and trains a domain adaptation model for predicting spatial cell compositions. The method was applied in brain and breast cancer tissues and accurately predicted the topography of cell subpopulations. spSeudoMap may help clarify the roles of a few, but crucial cell types.

**Supplementary Information:**

The online version contains supplementary material available at 10.1186/s13073-023-01168-5.

## Background

Spatial transcriptomics has been widely adopted as a tool to explore genome-wide spatial RNA expression in various tissues [1]. It paves the way to thoroughly investigate the spatial context of cells and their interactions in an unbiased manner [2]. One of the limitations of spatial transcriptomics data is the fact that spots are not directly interpreted as cells. Therefore, multiple computational approaches have been suggested for accurate spatial mapping of cell types by integrating spatial and single-cell transcriptomics [3–12]. They can be further utilized to speculate the spatial infiltration pattern of a few cell types that play a key role in the pathophysiology of various diseases [13–16]. In this case, certain cell populations, such as immune cell subtypes, can be described in detail by jointly analyzing spatial data with single-cell RNA sequencing (scRNA-seq) data acquired from cell sorting strategies such as fluorescence-activated cell sorting (FACS) and magnetic-activated cell sorting (MACS) based on cell surface markers [17]. However, there is a major drawback in the practical usage of spatial mapping methods employing scRNA-seq data. The majority of the methods are based on the assumption that cell types and proportions are similar between the two transcriptomic datasets [3–11], and cell type-specific signatures defined from the single-cell data are translated to decipher spatial cell compositions. When the sorted scRNA-seq data are utilized as a reference, the data explain only part of the cell types from the spatial data. Besides, certain cell types can be depleted or enriched during the cell dissociation procedure [18], and cell composition may vary across the tissue acquisition sites. Therefore, the integration of single-cell and spatial datasets creates a bias in estimating the cellular fraction. There is a need for the development of a computational model that flexibly integrates scRNA-seq data of cell subpopulations with spatial transcriptomic data.

In this regard, we created a model, spSeudoMap, which utilizes sorted scRNA-seq data to create a cell mixture resembling the spatial data and predicts the spatial cell composition based on CellDART [10], a domain adaptation approach. More specifically, the cell types exclusively present in the spatial data, named the pseudotypes, and the cell types shared between the two transcriptomic data, named the target types, are defined. Then, the fraction and expression profiles of the pseudotypes in the cell mixture are assigned by referencing the spatial and single-cell datasets. The rest of the mixture, the portion for the target types, is filled with randomly sampled cells from single-cell data. As a result, the cell mixture and spatial transcriptomic data are jointly analyzed based on domain adaptation to obtain a spatial map of the cell subpopulation. Our approach adjusts the discrepancy of cell type and proportion in single-cell and spatial data and is capable of precisely estimating the spatial distribution of the minor cell types.

## Methods

### Composition of public datasets

#### Human brain cortex

Spatial transcriptomic datasets of postmortem human DLPFC tissues were acquired from a 30-year-old subject without neurological disorders. Two Visium slides, named “151673” and “151676”, which are 300 μm apart were selected for the analysis [19]. The count matrix (151673: 33,538 genes across 3639 spots; 151676: 33,538 genes across 3460 spots) and cortical layer information of the spatial spots were utilized. The spots were assigned to a specific cortical layer [ranging from layers 1 to 6 and white matter (WM)] based on the spatial expression pattern of layer-specific marker genes and the expert’s opinion, as described in the paper [20]. The spots for which the layer could not be determined were classified as not available (NA). For the integrative analysis, a single-nucleus dataset obtained from the DLPFC of postmortem healthy individuals was adopted [21]. The count matrix (30,062 genes across 35,212 cells) and the cell type annotations defined based on the well-known marker genes were used [22]. Among the 33 cell types, 10 layer-specific excitatory neurons (from Ex_1_L5_6 to Ex_10_L2_4) were selected. This subpopulation of the single-nucleus dataset was considered a simulation of scRNA-seq data acquired by physically sorting excitatory neurons and was provided as an input for spSeudoMap.

#### Mouse brain coronal section

The Visium spatial transcriptomic dataset of the mouse brain coronal section was obtained from a C57BL/6 mouse that was at least 8 weeks old. The dataset, named “V1_Adult_Mouse_Brain,” was downloaded from the data repository provided by 10x Genomics [23]. Additionally, single-nucleus data extracted from mouse brain coronal tissue were included in the joint analysis [24]. The brains of a female and a male C57BL/6 mouse, both 56 days old, were sectioned and processed for sequencing analysis. The count matrices were composed of 32,285 genes and 2702 spots for spatial data and 31,053 genes and 40,532 cells for single-cell data. The cell types of the single-cell data were defined using reported marker genes [25, 26] and an in situ hybridization dataset from the Allen Brain Atlas (https://mouse.brain-map.org/static/atlas) [27], as described in the paper [11]. Among the 59 cell types, 23 region-specific neuron types were selected, and the subpopulation of single-nucleus data was utilized for the generation of the cell mixture.

#### Human breast cancer

Both spatial and single-cell transcriptomics data for breast cancer were extracted from an 88-year-old female subject (ID: 4290) without a previous history of treatment [28–30]. The cancer tissue was invasive ductal carcinoma (IDC) with ER-positive (90%, 3+), PR-positive (30%, 2+), and HER2-negative (1+) profiles, and cancer had invaded the adjacent skin and chest wall (pathologic T stage: T4b). The single-cell data (5789 cells and 29,733 genes) from the same patient were composed of cancer or normal epithelial cells, perivascular-like (PVL) cells, cancer-associated fibroblasts (CAFs), endothelial cells, and immune cells. This unsorted data was considered reference datasets for the prediction of spatial cell composition. In addition, CD45+ sorted single-cell data (29,900 cells and 31,993 genes) acquired from ER-positive IDC patients (*n*=4, ID: BC1, BC2, BC4, BC6) were utilized [31, 32], and the spatial immune cell composition was estimated using spSeudoMap.

### Clustering and annotation of single-cell and spatial data

The clustering and visualization of transcriptomics datasets were implemented using Seurat (v.4.0.5) [33] in R (v.4.1.1). First, count normalization was performed, and the total count was set to 10,000 across all cells and spots. Then, the count matrices were natural log-transformed [ln(1 + *X*)], and the top 2000 highly variable genes (HVGs) were chosen by standardizing counts with the mean-variance relationship (vst method) [4]. Next, the matrices were regressed against the total count and scaled such that the mean and standard deviation for HVGs were 1 and 0, respectively. After reducing the dimensionality of the dataset using principal component analysis (PCA), the top 30 PCs were selected for the downstream analysis. A shared nearest neighborhood (SNN) graph was constructed based on pairwise cell-cell distances calculated on PC space. The Louvain community detection algorithm [34] was applied with a resolution of 0.5, and the resulting clusters were visualized with uniform manifold approximation and projection (UMAP) plots [35].

For human and mouse brain single-nucleus datasets, the cell annotation information offered by the paper was adopted and visualized on UMAP plots. In the unsorted human breast single-cell data, the cell annotation was originally obtained from the pooled single-cell data of all patients, and each cell type of one of the patients (ID: 4290) contained only a small number of cells. Therefore, the cells were reannotated based on the Louvain cell clustering results by an automatic cell type assignment tool [36] and cell type information from the paper [28]. Additionally, the cells from the CD45+ sorted breast dataset were annotated by the automatic cell type annotation method [36].

Meanwhile, for mouse brain spatial data, the spot clusters were named according to the corresponding anatomical structure (amygdala, caudoputamen, cortex, ependyma, hypothalamus, meninges, piriform cortex, thalamus, and white matter) defined in Allen Brain Reference Atlases (https://mouse.brain-map.org/static/atlas) [27].

### spSeudoMap: spatial mapping of the transcriptomics of the cell subpopulations

**Fig. 1:**
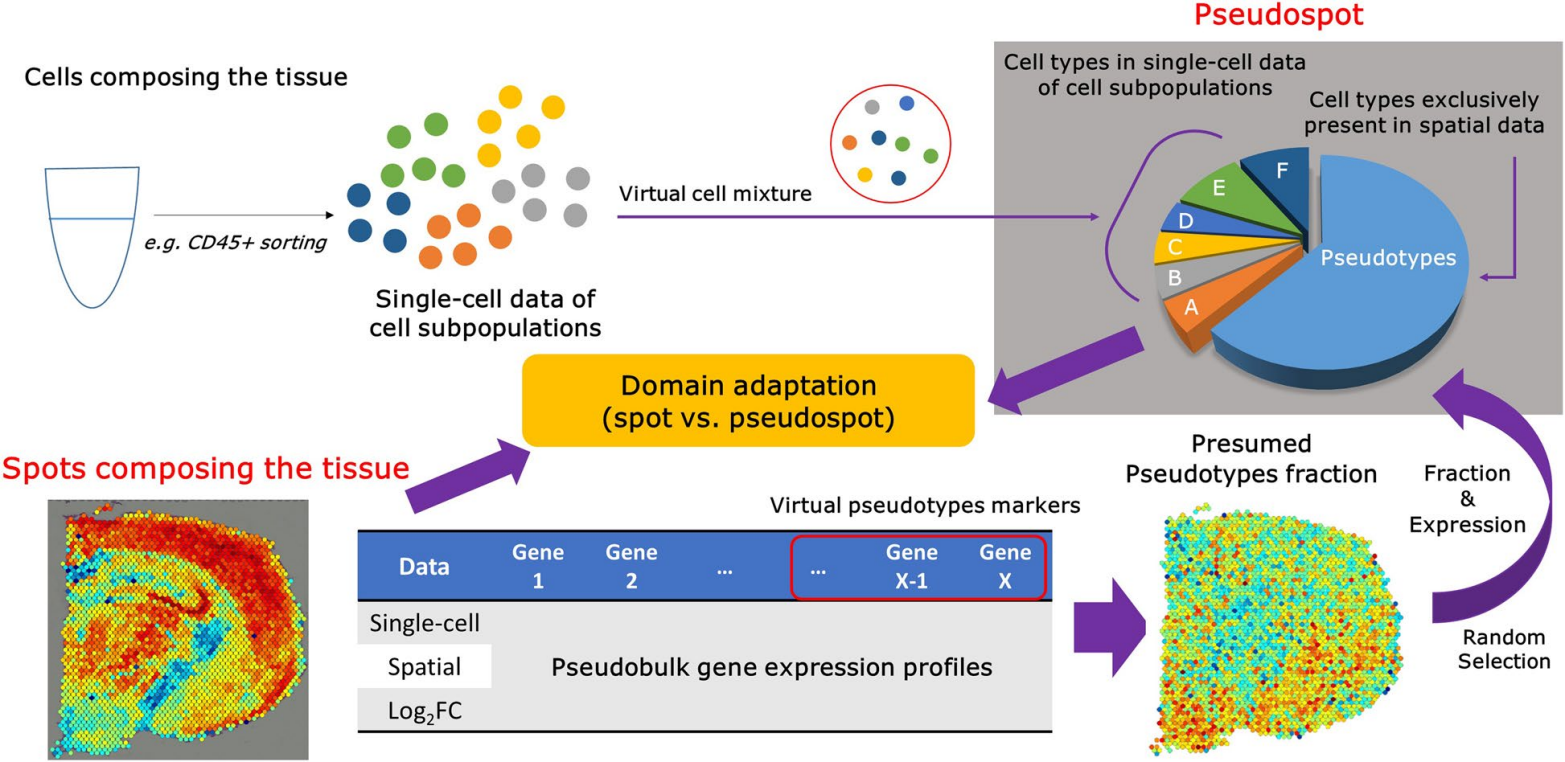
Mapping cell subpopulations to the spatial transcriptomic data with spSeudoMap. The cell types of the single-cell transcriptomic data acquired from cell sorting experiments can be spatially mapped to the tissue using spSeudoMap. The single-cell data of cell subpopulations are composed of sorted cells from the tissue, and the cell types cover only part of those in the spatial transcriptomics data. To create the reference dataset that mimics the spatial data, virtual cell mixtures, pseudospots, are defined in which all cell types from the tissue are included. First, the cell types exclusively present in the spatial data are aggregated and named pseudotypes. The virtual markers for the pseudotypes are selected from the top genes highly expressed in spatial pseudobulk compared to single-cell pseudobulk data. Then, the pseudotype fraction in the spatial spots is estimated from the module scores (sc.tl.score_genes in Scanpy) of the top 20 pseudotype markers. The fraction and gene expression of the pseudotypes are assigned based on the presumed pseudotype fraction and expression of a randomly selected spatial spot. Lastly, the target type proportion of the pseudospot, explained by cell types in the single-cell data, is filled with the randomly sampled cells from the single-cell data of cell subpopulations. Finally, the pseudospot is considered a reference dataset for the domain adaptation method CellDART [10]

To estimate a spatial map of cell types for single-cell data of cell subpopulations such as sorted or enriched single-cell datasets, a synthetic cell mixture that contains all cell types from spatial data is defined (Fig. 1). It is intended to create a reference dataset that is highly similar to the spatial transcriptomic data. For each mixture, the fraction of the exclusive cell types from the spatial data is assigned, and their synthetic gene expression profiles are created. The rest of the cell mixture is generated from the single-cell data. The process is implemented in Scanpy (v.1.5.1) [37] and Numpy in Python (v.3.7).

$${RC}_{sc}=\left(\begin{array}{ccc}{a}_{11}& \cdots & {a}_{1y}\\ {}\vdots & \ddots & \vdots \\ {}{a}_{x1}& \cdots & {a}_{xy}\end{array}\right),{RC}_{sp}=\left(\begin{array}{ccc}{b}_{11}& \cdots & {b}_{1v}\\ {}\vdots & \ddots & \vdots \\ {}{b}_{u1}& \cdots & {b}_{uv}\end{array}\right)$$

$${NC}_{sc}=\left(\begin{array}{ccc}{a}_{11}^{\prime }& \cdots & {a}_{1y}^{\prime}\\ {}\vdots & \ddots & \vdots \\ {}{a}_{x1}^{\prime }& \cdots & {a}_{xy}^{\prime}\end{array}\right),{NC}_{sp}=\left(\begin{array}{ccc}{b}_{11}^{\prime }& \cdots & {b}_{1v}^{\prime}\\ {}\vdots & \ddots & \vdots \\ {}{b}_{u1}^{\prime }& \cdots & {b}_{uv}^{\prime}\end{array}\right)$$

$${a}_{ij}^{\prime }=\left({a}_{ij}/\sum_{j=1}^y{a}_{ij}\right)\times \textrm{10,000},{b}_{ij}^{\prime }=\left({b}_{ij}/\sum_{j=1}^v{b}_{ij}\right)\times \textrm{10,000}$$

$$C=\left\{{c}_1,{c}_2,\cdots, {c}_h\right\}$$

RC_sc_: raw count matrix for single-cell data, RC_sp_: raw count matrix for spatial data, NC_sc_: total normalized single-cell count matrix, NC_sp_: total normalized spatial count matrix, *x*, *y*: number of cells and genes in single-cell data, *u*, *v*: number of spots and genes in spatial data, *C*: set of the index for overlapping genes between single-cell and spatial data. The gene index in set *C* is shared between the two transcriptomic data (for example, ${a}_{2{\textrm{c}}_1}$ and ${b}_{2{\textrm{c}}_1}$ indicate the counts of the same gene with index *c*_1_ in the second cell and spot, respectively).

First, a fixed number of cells (*n*; brain: 8 and breast: 10) and cell type annotations are randomly sampled from single-cell data with random weights, and a cell mixture named sub-pseudospot is created as with CellDART [10]. The cell types that overlap between the single-cell and spatial data are called “target types.” These cell types are assumed to be identical to the cell types from single-cell data. The Wilcoxon rank-sum test is performed, and the top *l* markers for each target cell type are pooled. A marker panel (total number of genes: *m*) is curated by extracting intersecting genes with the total gene list of spatial data. For each sub-pseudospot, the composite gene expression profiles of the marker panel are calculated.

$$\textrm{S}={\left({s}_i\right)}_{i=1}^n=\left({s}_1,{s}_2,\cdots {s}_n\right)\kern0.50em {s}_i\in \left\{1,2,\cdots x\right\}$$

$$T={\left({t}_j\right)}_{j=1}^m=\left({t}_1,t,\cdots {t}_m\right)\kern0.50em {t}_j\in C$$

$$F={\left({f}_i\right)}_{i=1}^n=\left({f}_1,{f}_2,\cdots {f}_n\right)\ \sum_{i=1}^n{f}_i=1$$

$$G={\left({g}_j\right)}_{j=1}^m=\left({g}_1,{g}_2,\cdots {g}_m\right)\kern0.50em {g}_j=\sum_{i=1}^n{f}_i{a}_{s_i{t}_j}^{\prime }$$

*n*: total number of the cells to be randomly sampled from the single-cell data, *m*: total number of marker genes from single-cell data: *S*: randomly selected index of the cells, *T*: index of the marker genes in the single-cell count matrix (RC_sc_), *F*: proportion of each sampled cell in the cell mixture, *G*: composite gene expression profiles of the cell mixture (named sub-pseudospot).

Next, the cell types present in the spatial data but absent in the single-cell data are aggregated, and the aggregate is named the “pseudotypes.” The pseudotype markers are extracted from pseudobulk analysis of both transcriptomic data. It can be assumed that the genes showing greater pseudobulk expression in spatial data than single-cell data are pseudotype markers. Therefore, the summed counts for each gene are divided by the total counts, and the normalized counts are compared between the two datasets. The ratio of the normalized counts between spatial to single-cell data is log_2_-transformed, and the genes are sorted in descending order of the log fold change. The top *k* genes not overlapping with the target type markers are selected as pseudotype markers, and the top 20 genes are used as predictors of the pseudotype fraction. The target types and pseudotype markers are combined and named the “composite marker panel.”

$$\begin{aligned} {BC}_{sc}=\left(p_j\right)_{j=1}^h=\left(\sum_{i=1}^xa_{ic_1},\cdots\sum_{i=1}^xa_{ic_h}\right)\\ {BC}_{sp}=\left(q_j\right)_{j=1}^h=\left(\sum_{i=1}^ub_{ic_1},\cdots\sum_{i=1}^ub_{ic_h}\right) \end{aligned}$$

$$\begin{aligned} logFC(j)=\log_2\left(\frac{q_j/\sum_{k=1}^hq_k}{p_j/\sum_{k=1}^hp_k}\right)\\ w=\underset{j\in Cj\not\in T}{\arg\;topk}\left[logFC(j)\right]=\left(r_1,r_2,\cdots,r_k\right)\end{aligned} $$

BC_sc_: pseudobulk count matrix for single-cell data, BC_sp_: pseudobulk count matrix for spatial data, logFC: log fold change between the pseudobulk counts, *w*: index number for the top *k* genes with the highest logFC.

Then, the pseudotype fraction in a given spot of the spatial data is presumed to be correlated with an enrichment score for the top 20 pseudotype markers (scanpy.tl.score_genes in Scanpy). The genes of spatial data are divided into 25 bins according to the log-normalized expression level. For each marker gene, a total of 50 control genes are selected from the same bin and pooled. The enrichment score is calculated by subtracting the average expression of control gene pools from that of pseudotype markers [38]. The distribution of a created module score is scaled to have a given mean (*M*^′^) and standard deviation (*σ*^′^). The values over 1 and less than 0 are replaced with 1 and 0, respectively. The scaled module score is considered as a pseudotype fraction of a spatial spot, assuming a linear relationship between the two.

$${MS}_{sp}={\left({e}_i\right)}_{i=1}^u$$

$$\begin{aligned} Z_{sp}= \left(\frac{MS_{sp}-M}\sigma\right)\sigma^{'}+M^{'} \\ Z_{sp}=\left(z_i\right)_{i=1}^u=\left\{\begin{array}{cc} 0& if \;Z_{sp}<0\\ 1& if\;Z_{sp}>1\\ Z_{sp} & otherwise \end{array}\right. \end{aligned}$$

MS_sp_: module scores for the top 20 pseudotype markers calculated in spatial data, *M*, *σ*: mean and standard deviation of MS_sp_, *M*^′^, *σ*^′^ : mean and standard deviation of the presumed pseudotype fraction in spatial transcriptomic spots, *Z*_sp_: presumed pseudotype fraction.

To create a reference dataset, a sub-pseudospot is aggregated to the pseudotype portion of a randomly chosen spot, and the combined gene expression for the composite marker panel is calculated (Additional file 1: Fig. S1). The resulting cell mixture is named the pseudospot. Since pseudotype markers are selected according to the log fold change in the pseudobulk approach, the expression of pseudotype markers in pseudotypes is expected to be significantly higher than that of target type markers. Additionally, for simplicity, the expression of all pseudotype markers in a spot is assumed to be directly proportional to that in the pseudotypes of the spot. Thus, the target type marker expression in pseudotypes is set to 0, and the pseudotype marker expression is assigned by multiplying the normalized count of the selected spot by the presumed pseudotype fraction.

$$\begin{aligned} T^{'}=\left(t_j^{'} \right)_{j=1}^{m+k}=\left\{\begin{array}{lc}t_j&if\;j\leq m\\ r_{j-m}&otherwise\end{array}\right.t_j^{'}\in C \end{aligned}$$

$$\begin{aligned} G^{'}=\left(g_j^{'}\right)_{j=1}^{m+k}=\left\{\begin{array}{lc}\left(1-z_\beta\right)g_j&if\;j\leq m\\z_\beta b_{\beta t_j^{'}}^{'}+\left(1-z_\beta\right)\sum_{i=1}^nf_ia_{s_it_j^{'}}^{'}&otherwise\end{array}\right.\end{aligned}$$

*T*^′^: index of the marker genes and pseudotype markers in the single-cell count matrix (RC_sc_), *G*^′^: composite gene expression profiles of the modified cell mixture (named pseudospot), *β*: randomly selected index of the spot

Finally, the gene expression profiles of the pseudotypes and the sub-pseudospot are summed with the pseudotypes to target type ratio as a weight, and the integrated expression of a pseudospot is obtained (Additional file 1: Fig. S1). Of note, the pseudotype fraction is extracted from the same spatial spot that the expression profile is referenced. The algorithm for generating a pseudospot is summarized with the above formulae. The pseudospots and spatial transcriptomic data are jointly provided as inputs for the domain adaptation in CellDART [10].

### Performance evaluation of spSeudoMap

The capability of spSeudoMap to predict the spatial composition of cell subpopulations was assessed in the three different datasets. In human DLPFC tissue, the accuracy of the model to localize 10 layer-specific excitatory neuron types to corresponding cortical layers was measured by receiver operating characteristic (ROC) analysis. The layer annotation of each spatial transcriptomic spot (layers 1 to 6 and WM) offered by the paper was considered a gold standard of spot identity [20]. The area under the ROC curve (AUROC) was calculated to explain the performance across all neuron types. For instance, a fraction of Ex_3_L4_5, the cell type known to be highly localized in layers 4 and 5 is predicted in all spots, and the “sensitivity” and “1 – specificity” of classifying a spot as belonging to layers 4 and 5 are plotted for every threshold value. The AUROC is calculated and it represents the performance of the model in Ex_3_L4_5. In the case of the mouse brain sample, the spot clusters were named after the anatomical region, and the spatial distribution of region-specific excitatory neuron types across the clusters was examined. To further assess the performance of spSeudoMap in brain tissues, the spatial distribution of pseudotype fraction estimated from spSeudoMap was compared with the spatial expression pattern of missing cell type markers. The missing cell type markers were discovered by performing differential gene expression analysis between excitatory neurons and others in unsorted single-cell data containing whole cell types. Wilcoxon rank-sum test was implemented and the Bonferroni method was applied for multiple comparison corrections. The top *k* (the number of pseudotype markers) genes showing the highest log_2_ fold change were selected and the module score was calculated. Spearman’s correlation coefficient was computed between the predicted pseudotype fraction and the module score across all spots. Finally, in human breast tissue, the spatial localization patterns of the top immune cell subpopulation were mapped. The spatial correlation patterns were evaluated by Spearman’s correlation coefficient and visualized with a heatmap.

The performance of spSeudoMap was compared in two different aspects. First, the spSeudoMap was tested whether it is superior to existing cell type decomposition methods (CARD, CellDART, Cell2location, DSTG, RCTD, and SPOTlight) in mapping the cell types in the situation that single-cell transcriptomic data only has subpopulation of cell types. The count matrix for selected cell types from the single-cell dataset of human DLPFC was provided as an input for the decomposition tools. CARD predicts spatial cell composition based on non-negative matrix factorization (NMF) and considers spatial correlation by a conditional autoregressive model [12]. Spots with a total count of less than 100 and genes with the number of spots showing a non-zero count of less than 5 were excluded from the analysis. CellDART decomposes spatial cell proportions based on domain adaptation [10]. The numbers of sampled cells in the cell mixture were set to 8 and 10 in brain and breast cancer tissues, respectively. The number of markers per cell cluster was set to 20. Cell2location predicts spatial cell proportion based on the Bayesian statistical model [11]. The expected cell abundance in each spatial spot was set to 8 in the brain tissue. The number of iterations was 30,000, and default values were given to the rest of the parameters in the model. DSTG constructs a linked graph between the spatial data and cell mixture created from single-cell data and applies a graph neural network to estimate spatial cell fraction [9]. RCTD predicts spatial cell compositions using maximum-likelihood estimation based on Poisson distribution [6]. The doublet mode was set to “full mode” which does not limit the number of cell types in the spots. SPOTlight utilizes NMF with regression to estimate the spatial distribution of cell types [8]. A total of 100 cells were randomly sampled from each cell cluster in single-cell data to enhance the computational speed. For all methods, the default parameters suggested in the user guide were applied for analyses.

Second, cell subpopulation mapping results by spSeudoMap were compared with the reference spatial map of cell types. As a surrogate to the real spatial distribution, the reference map was defined as spatial cell composition predicted using the unsorted single-cell dataset covering all cell types. It was presumed that the closer the spatial distribution of cell types obtained from spSeudoMap is to the reference map, the higher the performance of spSeudoMap. CellDART and Cell2location were selected as methods for obtaining the reference map since they showed similar high accuracy in localizing the layer-specific excitatory neurons [10, 11].

### Exploration of optimal parameter range in spSeudoMap

The key parameters for spSeudoMap are the number of markers per single-cell cluster (*n*), the ratio of the total number of single-cell to pseudotype markers (*m/k* ratio), and the mean and standard deviation of the presumed pseudotype fraction in spatial spots (*M*^′^ and *σ*^′^). The performance of the spSeudoMap was tested across the various parameters in human DLPFC datasets (slide number: 151676). Since the proportion of 10 layer-specific excitatory neurons was 0.53 among the single-cell data, *M*^′^ was set to 0.47. The cortical layer annotation in spatial data was used as a reference, and the layer discriminative accuracy of the predicted neuron fraction was assessed by AUROC. In general, spSeudoMap was capable of stably predicting the spatial distribution of neuron subpopulations with a median AUROC over 0.5 with *n* larger than 20, *m/k* larger than 1, and *σ*^′^larger than 0.05 (Additional file 1: Fig. S2). The corresponding parameter ranges were selected for the downstream analyses. For the human brain (slide 151673) and mouse brain tissues, *n* was set to 80, the *m/k* ratio to 4, and *σ*^′^to 0.1. In human breast cancer tissue, *n* was set to 40, the *m/k* ratio to 2, and *σ*^′^to 0.1. In brain tissues, *M*^′^ was assigned based on the proportion of non-excitatory neurons in the unsorted single-cell data containing all cell types and in breast cancer tissue, based on the proportion of non-immune cells (human brain: 0.47, mouse brain: 0.67, and breast cancer: 0.83). Other parameters for the domain adaptation were given as the user guidelines of CellDART [10].

## Results

### Scheme of spSeudoMap

spSeudoMap estimates the spatial map of the cell population by integrating spatial transcriptomic data with unmatched single-cell data which explains the subpopulation of cells in the tissue. The cell type and composition unmatch between the two datasets are modeled by creating a cell mixture, “pseudospot,” that contains not only cell types in single-cell data (“target types”) but also cell types exclusively present in spatial data (“pseudotypes”). Then, the proportion and expression of the pseudotypes are designated based on both transcriptomics data (Fig. 1). First, pseudobulk gene expression profiles are computed from both datasets. The top genes highly expressed in spatial transcriptomics data compared to single-cell data are selected as virtual pseudotype markers. These markers represent cell types that are not included in scRNA-seq data. Next, a pseudotype fraction in spatial spots is estimated by calculating module scores of pseudotype markers. Then, the pseudotype proportion and expression profiles in the cell mixture are designated by referencing the presumed pseudotype fraction and expression in a randomly selected spatial spot. The target cell type portion of the mixture is filled with randomly sampled cells from single-cell data. Finally, the pseudospot is considered a reference dataset for the domain adaptation model CellDART [10].

In contrast to CellDART, even when single-cell data only explains a portion of cell types in the tissue, spSeudoMap estimates information of missing cell type in single-cell compared to spatial transcriptomic datasets to create pseudospots that closely mimic real spatial spots. It is expected to minimize the domain shift between spatial data and pseudospots and maximize the performance of the domain adaptation suggested in CellDART.

### Mapping the excitatory neuron subpopulation in the human brain with spSeudoMap

**Fig. 2:**
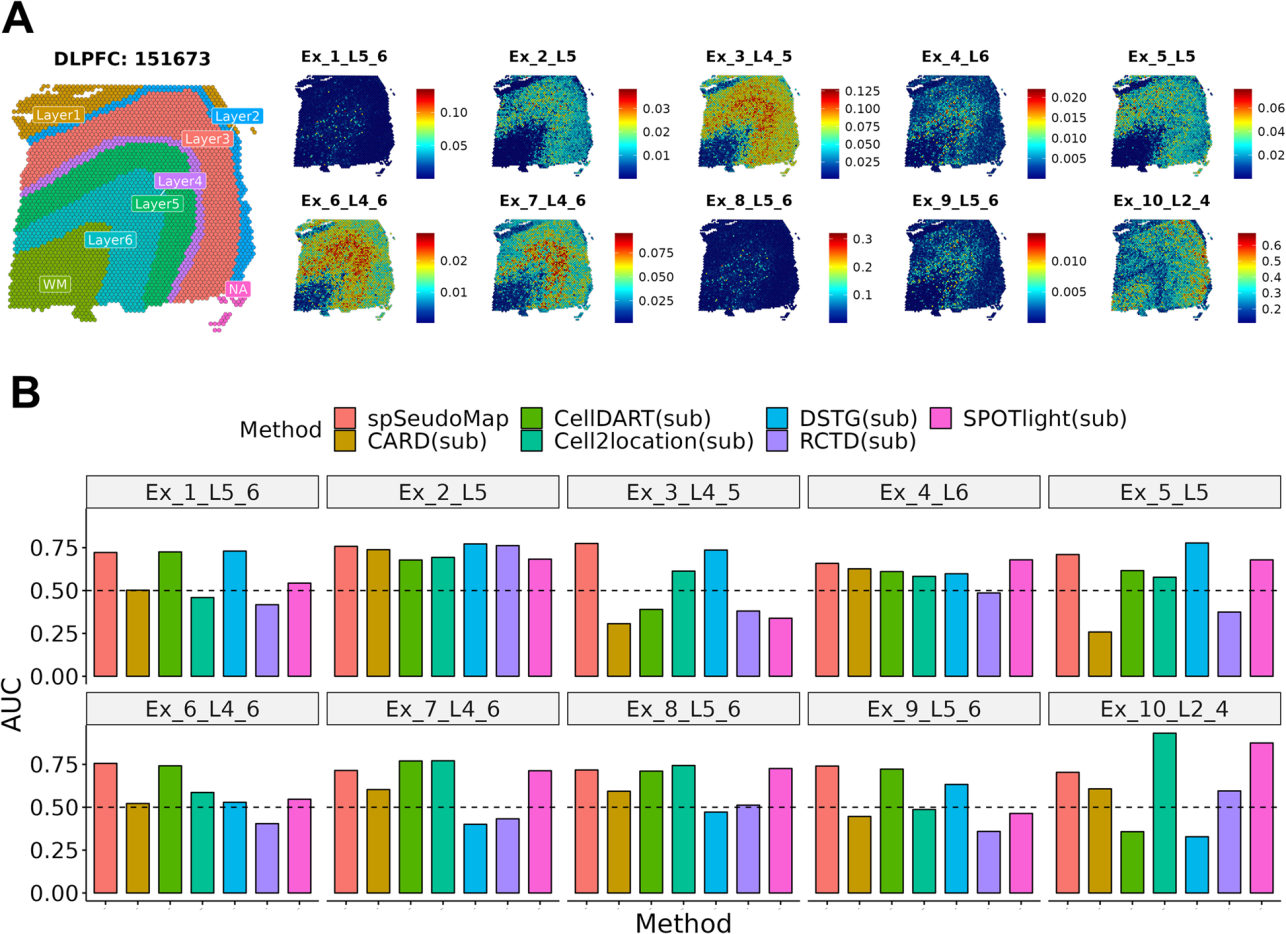
Decoding spatial maps of layer-specific excitatory neurons in the human brain. **A** The composition of ten excitatory neuron types in the human brain was predicted by spSeudoMap. The estimated cell fraction was spatially mapped to the tissue and visualized with colored bars. Overall, the spatial distribution of layer-specific neuron types was restricted to the corresponding cortical layer (layers 1 to 6). **B** The performance was measured by calculating the layer discriminative accuracy of the predicted layer-specific neuron fraction. It was represented by the area under the receiver operating characteristic curve (AUROC). The AUROC values of spSeudoMap in 10 cell subtypes were visualized with barplots and compared with those of CARD, CellDART, Cell2location, DSTG, RCTD, and SPOTlight. All methods were implemented using the simulated single-cell data of excitatory neuron subpopulations. In contrast to other methods, spSeudoMap showed a more stable performance represented by AUROC over 0.5 across all neuron types

The capability of the method, spSeudoMap, was assessed in the Visium spatial transcriptomic data of the human dorsolateral prefrontal cortex (DLPFC). First, 10 layer-specific excitatory neuron types were selected from the single-cell data of the human brain (for convenience, single-nucleus RNA-seq data were also called single-cell data) (Additional file 1: Fig. S3), and the cell subpopulation was jointly analyzed with the spatial data (slide no. 151673). Notably, the physical sorting of excitatory neurons did not precede scRNA-seq. Instead, for simulation purposes, excitatory neuron subpopulations were manually selected from the whole scRNA-seq data, and the cell types were mapped to the spatial transcriptomic data. Overall, the cell types were highly localized to the corresponding cortical layers (e.g., layers 4 to 5 for Ex_3_L4_5), although Ex_1_L5_6 and Ex_8_L5_6 showed uneven distribution patterns within the same layer (Fig. 2A).

### Performance stability of spSeudoMap in the human brain

The capability of spSeudoMap to stably predict the spatial map of cell subpopulations using single-cell data with varying cell composition. To test whether spSeudoMap is stable even when the composition of single-cell data deviates from that of spatial data, cells are randomly sampled from the excitatory neuron subpopulation of single-cell data with unequal probabilities. The number of cells in each cell type is altered within the range of ±30% and the probability of a cell being selected is assigned proportionally to the modified cell numbers of the cell types. Also, the number of cells sampled from the single-cell dataset (*n*) is modified (4, 8, 16, and 32) to test the stability of the performance. It is to mimic the condition in which certain cell types are depleted or enriched due to cell dissociation or to simulate spatial heterogeneity of the tissue [18]. The stability was tested for 20 different cell sampling probabilities in each *n* value. When *n* was from 4 to 32, which is a plausible range of the number of cells in each spatial transcriptomic spot, the layer discriminative accuracy of predicted cell fraction represented by median AUROC was above 0.5 for all excitatory neuron types (Additional file 1: Fig. S4). In summary, spSeudoMap presented stable performance although cell composition and diversity in single-cell data were altered across the wide range.

### Comparison across cell type mapping tools using subpopulations in the human brain

The 10 excitatory neuron subpopulation of single-cell data and spatial data were integrated using spSeudoMap, CARD, CellDART, Cell2location, DSTG, RCTD, and SPOTlight tools, and the spatial composition of neurons was predicted (Additional file 1: Fig. S5). It was intended to compare the performance of spSeudoMap with the existing cell type deconvolution methods when there is a mismatch of cell types between both transcriptomic datasets. First, in CellDART, Ex_3_L4_5 and Ex_10_L2_4 had lower cell fractions in the corresponding cortical layers than in other layers. Second, in Cell2location, Ex_1_L5_6 and Ex_9_L5_6 had opposite patterns of distribution, with higher cellular abundance in layers other than 5 and 6. Third, in SPOTlight, Ex_3_L4_5 and Ex_9_L5_6 revealed nonspecific patterns of spatial distribution. Last, in CARD, DSTG, and RCTD, more than half of the cell types showed lower cell fractions in the expected layers than in other locations.

To measure the layer discriminative accuracy of the predicted cell fraction, an AUROC was calculated, considering layer annotation as a reference. The AUROC was compared across the seven methods (Fig. 2B), and spSeudoMap showed superior performance than CARD, CellDART, Cell2location, DSTG, RCTD, and SPOTlight in 100, 80, 70, 70, 90, and 70% of cell types. In summary, within the optimal parameter range, spSeudoMap presented more stable performance across all cell types in the neuron subpopulation compared to other existing computational methods.

### Deciphering the excitatory neuron composition in the mouse brain with spSeudoMap

The performance of spSeudoMap was evaluated in coronal sections of the mouse brain (Additional file 1: Fig. S6). First, the two cell type deconvolution tools, CellDART and Cell2location, which showed comparable high performance in the previous study [10], were tested on the original single-cell data containing all cell types (Additional file 1: Fig. S7A). The two prediction results were considered reference standards for assessing spSeudoMap. In both methods, the region-specific neurons showed spatially restricted patterns according to their expected spatial predominance (Additional file 1: Fig. S8).

**Fig. 3:**
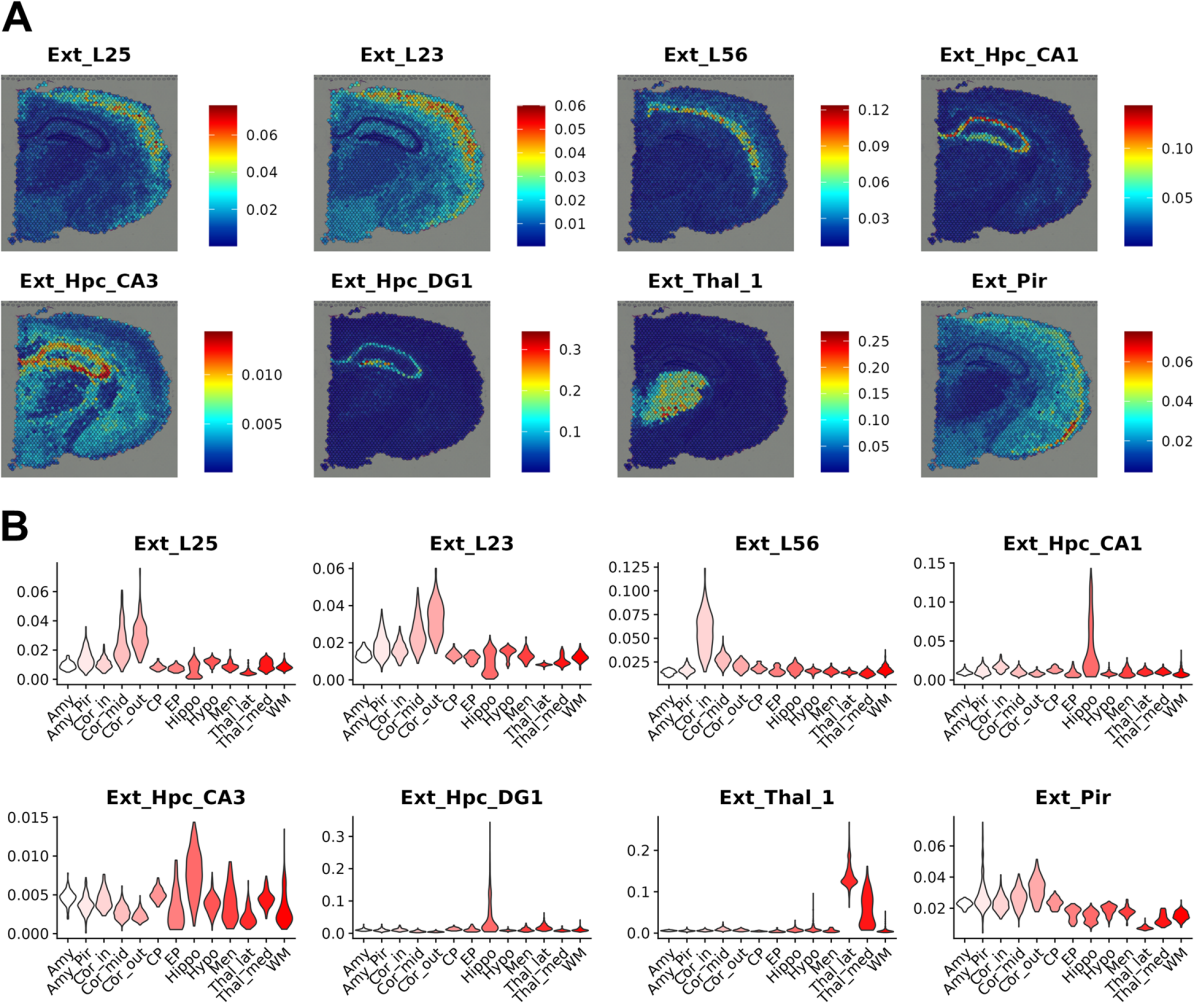
Predicting the spatial composition of region-specific neuron types in the mouse brain. **A** The proportion of the representative region-specific neuron types was estimated by spSeudoMap and mapped to the mouse brain. Overall, the 8 neuron types presented were predominantly localized to the corresponding anatomical locations. **B** Spatial spots were clustered based on their gene expression profiles, and the spot clusters were named after the anatomical locations. The predicted neuron fraction was highly distributed according to anatomical location. Ext_L25 to the mid-cortical layer, Ext_23 to the outer cortical layer, Ext_L56 to the inner cortical layer, Ext_Hpc_CA1, Ext_Hpc_CA3, and Ext_Hpc_DG1 to the hippocampus, Ext_Thal_1 to the thalamus, and Ext_Pir to the amygdala or piriform cortex area. Amy: amygdala, Amy_Pir: amygdala or piriform cortex, Cor_out: outer cortex, Cor_mid: mid cortex, Cor_in: inner cortex, CP: caudoputamen, EP: ependyma, Hippo: hippocampus, Hypo: hypothalamus, Men: meninges, Thal_lat: lateral thalamus, Thal_med: medial thalamus, and WM: white matter

The excitatory neuron subpopulation was selected as a simulation of sorted scRNA-seq data (Additional file 1: Fig. S7B) and spatially mapped to the brain with spSeudoMap (Fig. 3A). The 8 cell types showed a highly restricted distribution with a similar spatial predominance as the reference results (Fig. 3B and Additional file 1: Fig. S9). Other cell types were also mapped on the mouse coronal section data and compared with the reference results (Additional file 1: Fig. S10). Some of the neuron types having a low fraction (<0.05) estimated by spSeudoMap were distributed not only in the expected regions but also in the regions outside of interest. According to the results, spSeudoMap was capable of precisely predicting the spatial compositions of the main cell types composing the cell subpopulation.

### Elucidating cellular heterogeneity in human breast cancer with spSeudoMap

**Fig. 4.**
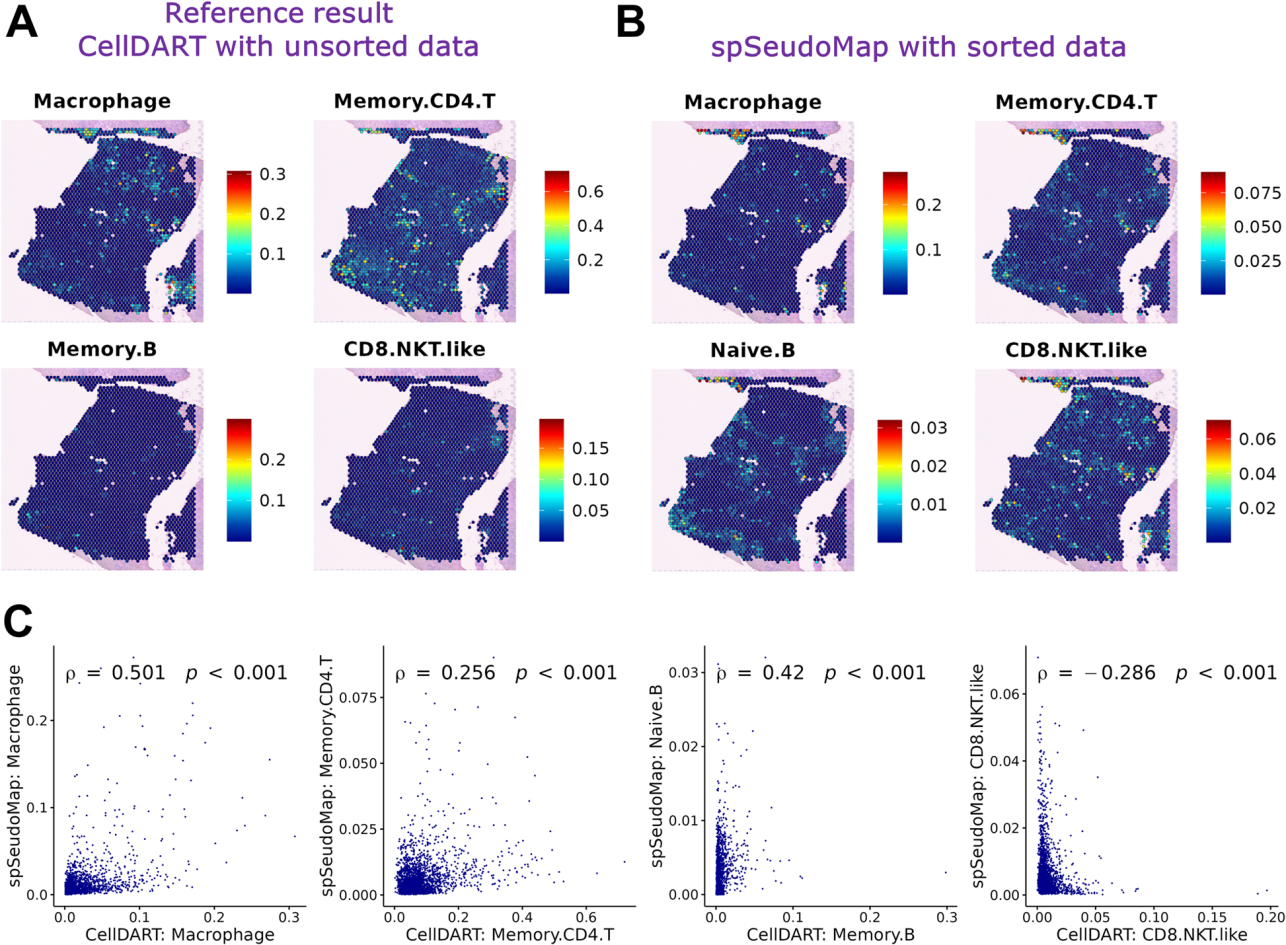
Exploring the spatial heterogeneity of immune cells in human breast cancer tissue. **A** The spatial composition of immune cells in human breast cancer was predicted by integrating spatial data with single-cell data covering all cell types. The results from CellDART were considered a reference for comparison with spSeudoMap. **B** The spatial composition of the immune cell types was predicted by spSeudoMap using CD45+ sorted single-cell data and visualized on the tissue. Macrophages (sum of macrophage_1, macrophage_2, and macrophage_3), memory CD4 T cells, and B cells showed similar spatial distributions, while CD8 NKT-like cells presented different patterns. **C** The scatter plots show the correlation between the cellular proportion predicted by spSeudoMap with sorted single-cell data and that estimated by CellDART with an unsorted single-cell dataset covering all cell types (reference). Spearman’s correlation coefficients and statistical significance (*p*-value) were calculated and are presented in the top-left corner of each plot. The correlation between CellDART and spSeudoMap was the highest in macrophages (the sum of macrophage_1, macrophage_2, and macrophage_3). Other cell types also showed weak but positive correlations except for CD8+ NKT-like cells, which showed a negative correlation

spSeudoMap was assessed in breast cancer tissue, which has a high level of heterogeneity. We mapped cell types of spatial transcriptomics of human breast cancer using two different scRNA-seq datasets: one dataset with unsorted whole-cell types and another dataset obtained after CD45+ cell sorting. The prediction results of CellDART obtained using all cell types in the unsorted single-cell data were considered a reference (Additional file 1: Fig. S11A). Immune cell types from CD45+ sorted single-cell data (Additional file 1: Fig. S11B) were spatially mapped to human breast cancer tissue using spSeudoMap. For macrophages, which are the immune cell type with the highest proportion in the tissue (Additional file 1: Fig. S11B), the spatial cell compositions were similar between spSeudoMap and the reference results from CellDART (Fig. 4A, B). Additionally, CD4+ T cells and B cells showed similar spatial patterns with positive spatial correlation (Fig. 4C), although B cells in the two single-cell datasets indicated different cell subtypes. However, the spatial localization patterns of CD8+ NKT-like cells, one of the minor cell types in scRNA-seq data, were different between spSeudoMap and the reference. Finally, to investigate the spatial interaction between the immune cells, the pairwise correlations between the cell fractions predicted by spSeudoMap were computed and visualized with a heatmap (Additional file 1: Fig. S12). Spatial correlation patterns were highly similar between one of the macrophage subtypes and memory CD4+ T cells. Additionally, high proximities were observed between another subtype of macrophages and CD8+ NKT-like cells. These findings implicate the spatial interaction of macrophages with T cells in the tumor tissue [39]. In short, spSeudoMap can be applied to decipher complex tumor microenvironments and to understand spatial interactions between cell subpopulations.

## Discussion

Exploring the spatial composition of infiltrating cells in tissues provides a key to understanding the molecular mechanism underlying the functional changes. In recent years, the crosstalk between immune cells and cancer cells has been highlighted as a major player in the development of tumor microenvironments [14]. Additionally, the complex interactions between the peripheral and central immunities are considered crucial for the progression of various neuroinflammatory diseases [15]. As a breakthrough to the research questions, the integration of spatial transcriptomics with the single-cell data of cell subpopulations can provide a cellular landscape of the few cell types, such as immune cells, in highly heterogeneous tissues.

Compared to the existing approaches, spSeudoMap, which is specialized in tracking the spatial distribution of subclusters of specific cell types, is more appropriate for capturing the transcriptomic changes in a few cell populations. The main contribution of the method is that it enables the mapping of cell subtypes explained in sorted scRNA-seq data to spatial transcriptomic data based on the pseudobulk approach of modeling the missing information in single-cell data. First, spSeudoMap was assessed in brain tissues, and the main region-specific excitatory neuron types could be accurately mapped according to anatomical locations (Figs. 2 and 3). Particularly in human brain tissue, the prediction results were stable even when the cell compositions deviated in single-cell data (Additional file 1: Fig. S4). The application of spSeudoMap could be extended to human breast cancer tissue to estimate the spatial distribution of immune cell types (Fig. 4). The predicted spatial patterns of the major immune cell populations were well correlated with the reference results, in which original single-cell data with all cell types were utilized for integration.

One of the key features of spSeudoMap is that it assumes the virtual cell types, pseudotypes, which are absent in single-cell data but present in the spatial data. Then, it finds markers of the pseudotypes by considering single-cell and spatial data as bulk sequencing datasets (Fig. 1). Moreover, spSeudoMap predicts the pseudotype fraction in each tissue domain and adopts the information to model the reference dataset, pseudospots. These processes along with domain adaptation estimate the missing information in single-cell transcriptomic data, thereby correcting the cell types and composition mismatch between single-cell and spatial data. This capability of the spSeudoMap could be further validated in brain datasets. The unsorted single-cell datasets from the brain contain whole cell types in the tissue, and missing cell type markers can be acquired by finding differential expressed genes between excitatory neurons and other populations. The spatial enrichment patterns of missing cell type markers of the single-cell data showed a positive correlation with the pseudotype fraction predicted by spSeudoMap (Additional file 1: Fig. S13). In contrast to spSeudoMap, most of the existing methods utilize cell type signatures solely extracted from the single-cell datasets of cell subpopulations, and it will be biased to select the genes differentially expressed inside of the cell subset. Thus, the signatures would not well represent the real expression profiles of cell types in the tissue. This may result in an imprecise estimation of the cellular composition. In this regard, some layer-specific excitatory neuron types showed nonspecific distribution when the single-cell data of cell subpopulations was directly given as an input to the existing methods (Additional file 1: Fig. S5). Although CARD utilizes spatial correlation of cell composition and adjusts the mismatch between single-cell and spatial data, many of the neuron types did not show layer-specific patterns of distribution.

As in the aforementioned simulation examples (Figs. 2 and 3), spSeudoMap can be applied to investigate the spatial compositions of the cell subtypes of a certain cluster. When the original single-cell data are composed of finely defined clusters and the clusters are closely located in terms of gene expression, their marker genes will largely overlap. It may interfere with the precise computation of cell fractions with the existing deconvolution tools. In that case, spSeudoMap can be applied as an alternative, and the selected cell subtypes and their count matrix are provided as inputs to the model. Furthermore, spSeudoMap can be directly applied to enriched single-cell data in which certain cell types are collected from multiple samples by cell sorting strategies, e.g., immune cells using CD45+ sorting. Otherwise, cell types that are not of interest are removed during data processing and specific clusters of single-cell data are selected to create the spatial map of the specific cell types.

There are additional considerations when implementing spSeudoMap to predict the spatial composition of the cell subpopulation. Among the main parameters of the model, the mean of the presumed pseudotype fraction (*M*^′^) must be given to generate pseudospots. For the single-cell data obtained from the cell sorting experiments, *M*^′^ can be considered a fraction of the negative population during the sorting. Alternatively, *M*^′^ can be determined based on the literature evidence that explains the cell type proportion. In fact, spSeudoMap is a generalized form of CellDART, and it flexibly models various situations. If the single-cell and spatial transcriptomic datasets have highly similar cell types, the mean and standard deviation of the pseudotype fraction can be set to 0, and then spSeudoMap is identical to CellDART. When both datasets have significantly different cell types, only the shared cell types can be selected, and their proportions and expression profiles can be given as inputs. In that case, *M*^′^ will be closer to 1 than 0. Meanwhile, in spSeudoMap, the distribution of the pseudotype fraction is assumed to be linearly proportional to the gene set enrichment score of the top pseudotype markers. Although the real distribution may be different, the domain adaptation process could manage the discrepancy between the pseudospots and spatial datasets (Additional file 1: Fig. S13). In addition, there may be a concern that the pseudotype markers extracted by the pseudobulk approach may contain nonspecific housekeeping genes which account for a major proportion of the total count in the tissue. When the Gene Ontology (GO) analysis was performed for the estimated pseudotype markers in brain and breast cancer tissues [40, 41], the markers were not associated with housekeeping gene sets such as cell proliferation and metabolism (Additional file 1: Fig. S14). In addition, since the pseudotype marker is used to determine approximately how much proportion the cell types of single-cell data occupies in a spot, the purpose is to estimate the approximate amount of the “target cell types” rather than to identify the genes not included in single-cell data. Last, when the rare cell types are mapped to the tissue, the cellular fraction may be overestimated in the regions outside those of interest, and the region-specific localization patterns may be less prominent. In addition, when the single-cell data contain additional cell types compared to the spatial data, as in the basophils and effector CD8 T cells in sorted single-cell data of breast cancer (Additional file 1: Fig. S11B), the prediction of minor immune cell fraction may be affected. Thus, caution is required when interpreting the results for the rare cell population.

## Conclusions

The spSeudoMap is a robust model to estimate the spatial configuration of cell subtypes explained by sorted scRNA-seq data in tissues with a high level of heterogeneity. It can be further utilized to describe the perturbation of complex intercellular interactions during disease progression, therefore capturing the spatial dynamics of pathophysiology.

## Supplementary Information

**Additional file 1: Fig. S1.** The schematic diagram for creating pseudospots, the reference dataset for the domain adaptation. **Fig. S2.** Exploration of optimal parameters for spSeudoMap. **Fig. S3.** Single-nucleus datasets for the human DLPFC tissue. **Fig. S4.** The performance stability of spSeudoMap. **Fig. S5.** Spatial distribution patterns of layer-specific neurons estimated by existing cell type deconvolution tools. **Fig. S6.** Spot clustering of mouse brain spatial transcriptome. **Fig. S7.** Single-nucleus data for the mouse brain coronal section. **Fig. S8.** Spatial maps of region-specific neurons in mouse brain using original single-cell data containing all cell types: the representative neuron types. **Fig. S9.** Distribution of the neuron subtypes in mouse brain across the locations predicted from single-cell data covering all cell types. **Fig. S10.** The spatial landscape of region-specific neurons in mouse brain: rest of the neuron types. **Fig. S11.** Single-cell data for the human breast cancer. **Fig. S12.** Spatial correlation patterns between immune cells in the human breast cancer tissue. **Fig. S13.** Comparison between spatial enrichment patterns for marker genes of missing cell types in single-cell data and the pseudotype fraction predicted from spSeudoMap. **Fig. S14.** Functional implication of pseudotype markers extracted from spSeudoMap.

## Data Availability

Publicly available transcriptomic datasets were utilized for the analyses. First, human DLPFC single-nucleus and spatial transcriptomic (slide no. 151673 and 151676) datasets can be downloaded from http://research.libd.org/spatialLIBD/ [19] and GSE144136 (https://www.ncbi.nlm.nih.gov/geo/query/acc.cgi?acc=GSE144136) [21], respectively. Second, mouse brain coronal section single-nucleus and spatial datasets can be accessed from E-MTAB-11115 (https://www.ebi.ac.uk/biostudies/arrayexpress/studies/E-MTAB-11115) [24] and 10x Genomics data repository (https://www.10xgenomics.com/resources/datasets) [23], respectively. Lastly, matched single-cell and spatial data from the breast cancer patient can be downloaded from GSE176078 (https://www.ncbi.nlm.nih.gov/geo/query/acc.cgi?acc=GSE176078) [29] and https://zenodo.org/record/4739739 [30], while CD45+ sorted single-cell data from GSE114727 (https://www.ncbi.nlm.nih.gov/geo/query/acc.cgi?acc=GSE114727) [32]. Python source code and R wrap function for spSeudoMap is uploaded on https://github.com/bsungwoo/spSeudoMap.
