# Supplementary material for: spSeudoMap: cell type mapping of spatial transcriptomics using unmatched single-cell RNA-seq data
Source: Genome Med. 2023 Mar 17;15:19. doi: 10.1186/s13073-023-01168-5 (PMC10021938; doi:10.1186/s13073-023-01168-5)
Supplement: Supplementary file 1 — Additional file 1: Fig. S1. The schematic diagram for creating pseudospots, the reference dataset for the domain adaptation. Fig. S2. Exploration of optimal parameters for spSeudoMap. Fig. S3. Single-nucleus datasets for the human DLPFC tissue. Fig. S4. The performance stability of spSeudoMap. Fig. S5. Spatial distribution patterns of layer-specific neurons estimated by existing cell type deconvolution tools. Fig. S6. Spot clustering of mouse brain spatial transcriptome. Fig. S7. Single-nucleus data for the mouse brain coronal section. Fig. S8. Spatial maps of region-specific neurons in mouse brain using original single-cell data containing all cell types: the representative neuron types. Fig. S9. Distribution of the neuron subtypes in mouse brain across the locations predicted from single-cell data covering all cell types. Fig. S10. The spatial landscape of region-specific neurons in mouse brain: rest of the neuron types. Fig. S11. Single-cell data for the human breast cancer. Fig. S12. Spatial correlation patterns between immune cells in the human breast cancer tissue. Fig. S13. Comparison between spatial enrichment patterns for marker genes of missing cell types in single-cell data and the pseudotype fraction predicted from spSeudoMap. Fig. S14. Functional implication of pseudotype markers extracted from spSeudoMap. [file 13073_2023_1168_MOESM1_ESM.pdf]

Additional file 1.

spSeudoMap: Cell type mapping of spatial transcriptomics using unmatched single-cell RNA-seq data

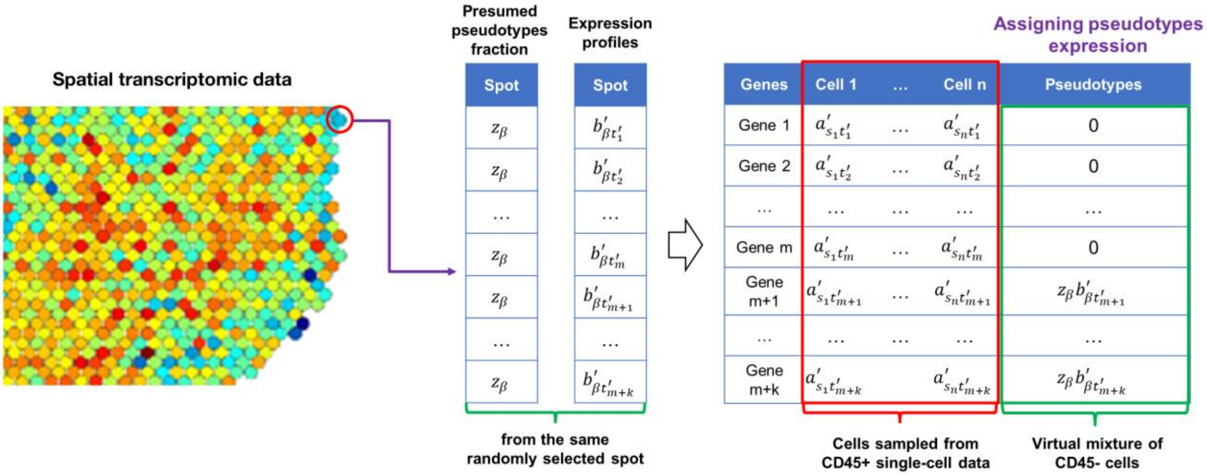

Fig. S1. The schematic diagram for creating pseudospots, the reference dataset for the domain adaptation

A spatial spot is randomly sampled from the spatial transcriptomic data. The presumed pseudotypes fraction (z_β) and expression profiles of the spot ($b'_{\beta t'_1}$, $b'_{\beta t'_2}$, $\cdots$ $b'_{\beta t'_{m+k}}$) are referenced. Then, the expression profiles of virtual pseudotypes markers (Gene m+1, ⋯ Gene m+k) in pseudotypes are assigned by multiplying the expression profiles of the selected spot with the pseudotypes fraction ($z_\beta b'_{\beta t'_j}$). The expression of the rest of the maker genes (Gene 1, ⋯ Gene m), the target types markers, in pseudotypes is set to 0. Finally, to create a pseudospot, a cell mixture that closely resembles the spatial spots, pseudotypes expression is aggregated with composite gene expression profiles of a cell mixture generated from a random sampling of cells from the single-cell data (e.g. CD45+ sorted single-cell RNA sequencing data).

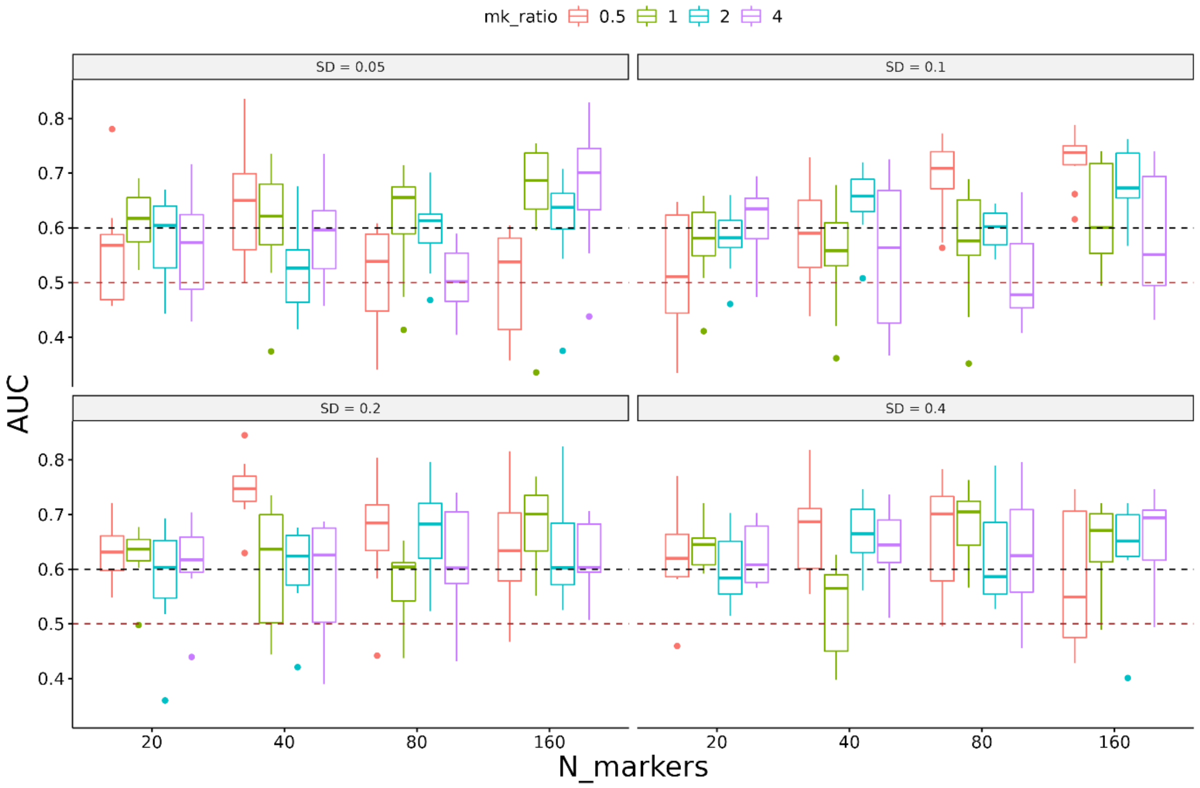

Fig. S2. Exploration of optimal parameters for spSeudoMap

The optimal parameter ranges for spSeudoMap were searched in human brain tissue (slide number: 151676). The main parameters composing the model, the number of markers per cell type (*n*; represented as N_markers in the plot), the ratio of the number of single-cell markers to pseudotypes markers (*m/k* ratio), and standard deviation of presumed pseudotypes fraction (σ'; represented as SD in the plot), were changed and the performance was evaluated. The 10 layer-specific excitatory neuron fraction predicted by spSeudoMap was assessed whether it precisely localizes the cell type to the corresponding cortical layer. Receiver operating characteristic analysis was implemented and the area under the curve (AUC) was calculated for each cell type. The AUCs across 10 neuron types were pooled and visualized with boxplots. The plot revealed that the performance of spSeudoMap was overall stable when n>20, m/k ratio>1, and σ'> 0.05.

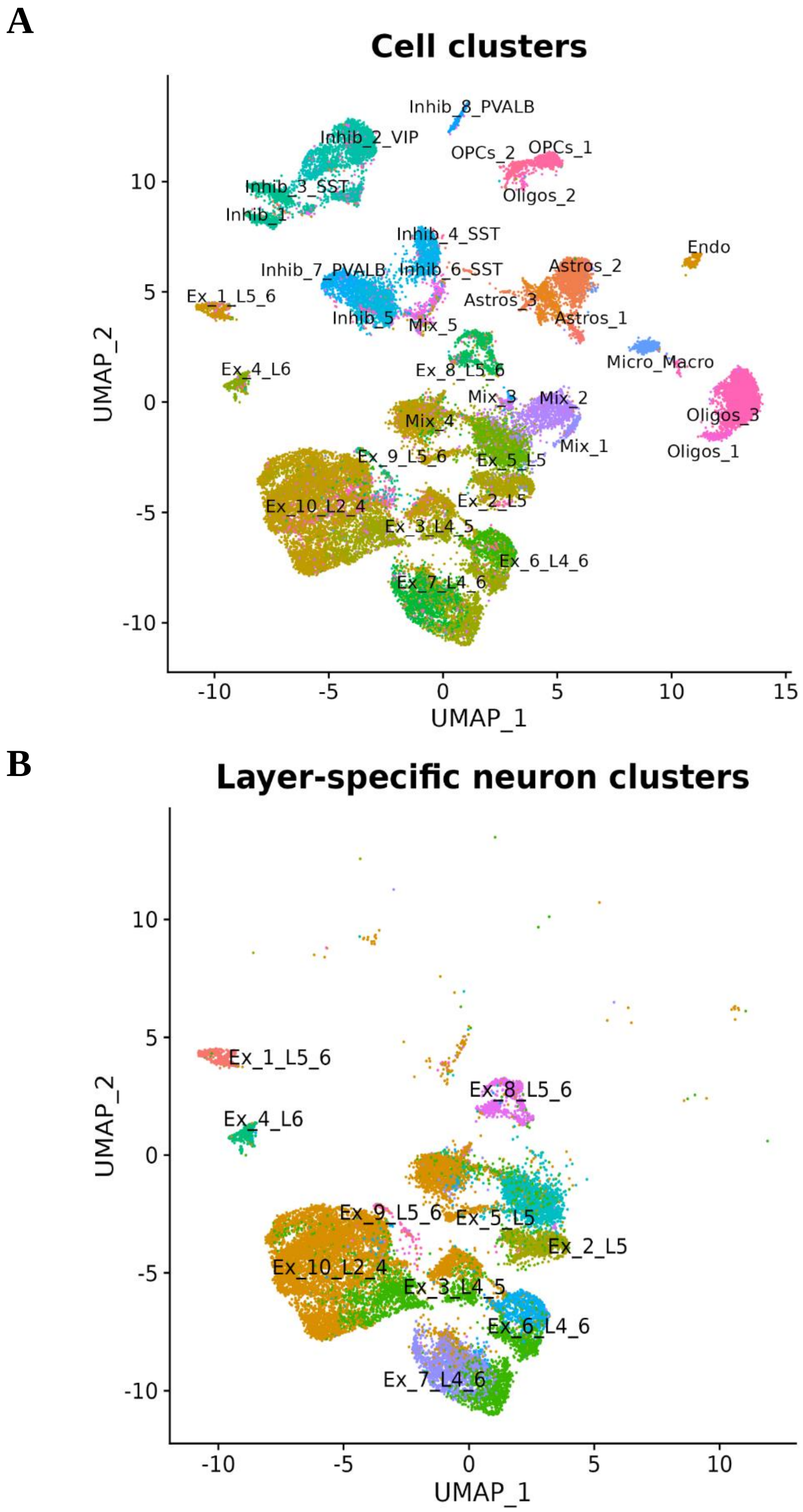

A
Cell clusters
Inhib_8_PVALB
Inhib_2_VIP
OPCs_2
OPCs_1
Inhib_3_SST
Oligos_2
Inhib_1
Inhib_4_SST
Endo
Inhib_7_PVALB
Inhib_6_SST
Astros_2
Ex_1_L5_6
Astros_3
Inhib_5
Mix_5
Astros_1
Micro_Macro
Ex_4_L6
Ex_8_L5_6
Mix_3
Mix_2
Mix_4
Oligos_3
Mix_1
Oligos_1
Ex_9_L5_6
Ex_5_L5
Ex_10_L2_4
Ex_2_L5
Ex_3_L4_5
Ex_6_L4_6
Ex_7_L4_6
UMAP_2
UMAP_1
B
Layer-specific neuron clusters
Ex_1_L5_6
Ex_8_L5_6
Ex_4_L6
Ex_9_L5_6
Ex_5_L5
Ex_2_L5
Ex_10_L2_4
Ex_3_L4_5
Ex_6_L4_6
Ex_7_L4_6
UMAP_2
UMAP_1

Fig. S3. Single-nucleus datasets for the human DLPFC tissue

(A) Dimensionality reduction was performed for the single-nucleus data and the cells were visualized on a uniform manifold approximation and projection (UMAP) plot based on their expression profiles. The cell type annotation was color-coded on the plot.

(B) Among the 33 cell types composing the brain, 10 layer-specific excitatory neurons were selected and utilized as the simulation dataset for the sorted single-cell data. The selected neuron types were visualized on UMAP.

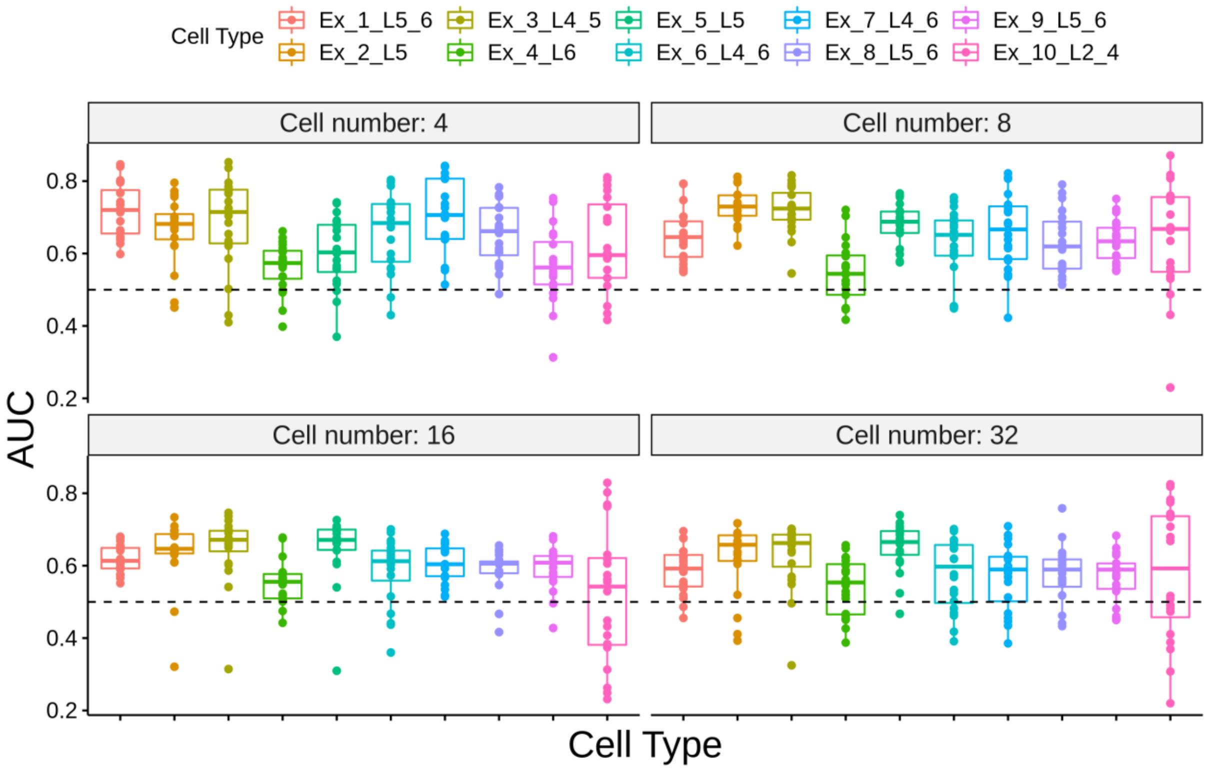

Fig. S4. The performance stability of spSeudoMap

The performance was tested by modifying the sampling probability of a cell and the number of cells to be sampled (*n*; represented as 'Cell number' in the plot) from single-cell data. The number of cells in each cell type was altered within the range of ±30% and the probability of cells being selected was modified according to the new number of cells in the cell types. The process was repeated 20 times for a wide range of *n* values (4, 8, 16, and 32). The performance was measured by computing layer discriminative accuracy of the predicted layer-specific neuron fraction. It was represented by the area under the receiver operating characteristic curve (AUROC) and the values across 10 neuron types were visualized with boxplots. AUROC values were highly consistent across all neuron types when *n* was from 4 to 32.

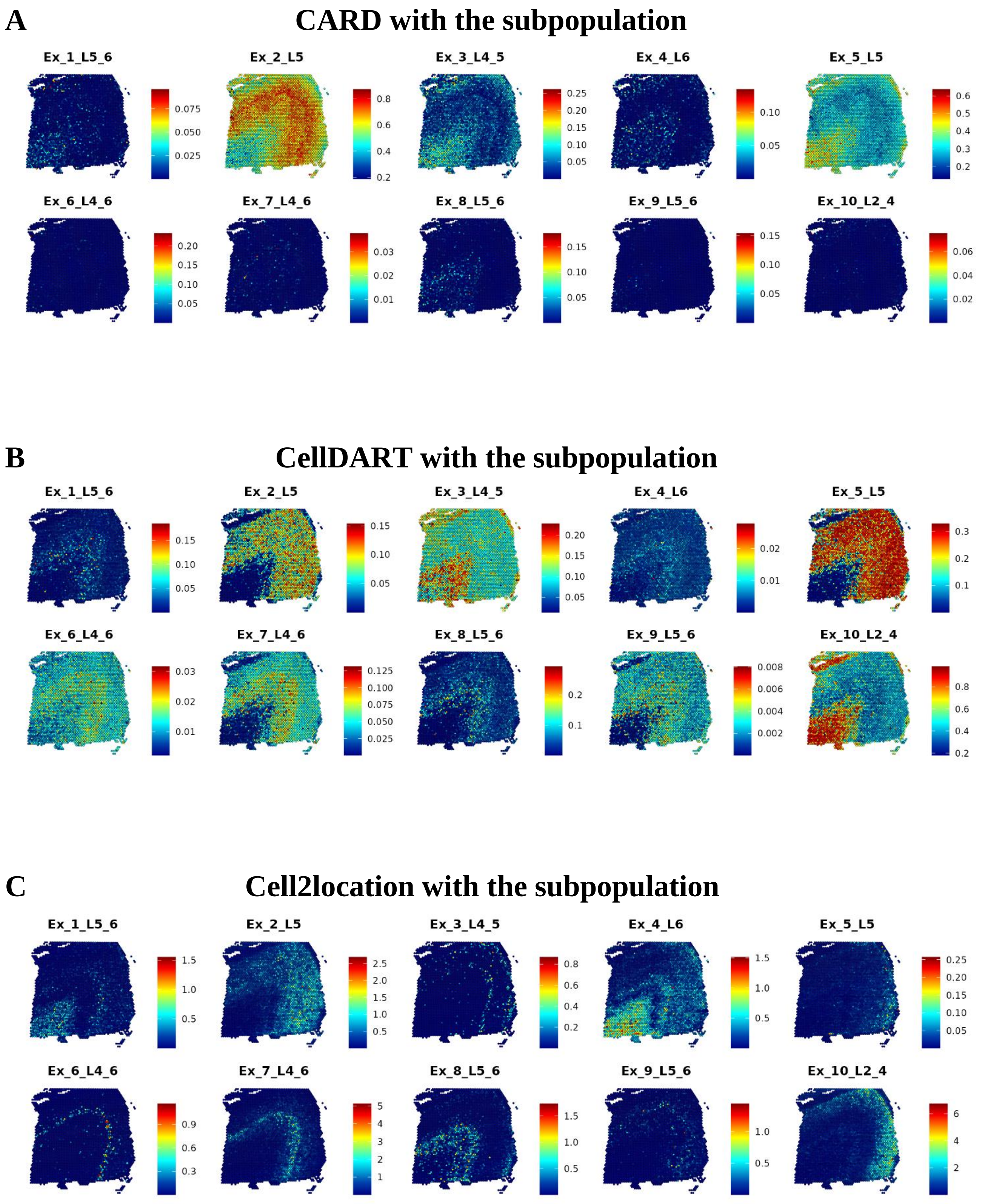
A
CARD with the subpopulation
Ex_1_L5_6
Ex_2_L5
Ex_3_L4_5
Ex_4_L6
Ex_5_L5
Ex_6_L4_6
Ex_7_L4_6
Ex_8_L5_6
Ex_9_L5_6
Ex_10_L2_4
B
CellDART with the subpopulation
Ex_1_L5_6
Ex_2_L5
Ex_3_L4_5
Ex_4_L6
Ex_5_L5
Ex_6_L4_6
Ex_7_L4_6
Ex_8_L5_6
Ex_9_L5_6
Ex_10_L2_4
C
Cell2location with the subpopulation
Ex_1_L5_6
Ex_2_L5
Ex_3_L4_5
Ex_4_L6
Ex_5_L5
Ex_6_L4_6
Ex_7_L4_6
Ex_8_L5_6
Ex_9_L5_6
Ex_10_L2_4

D **DSTG with the subpopulation**

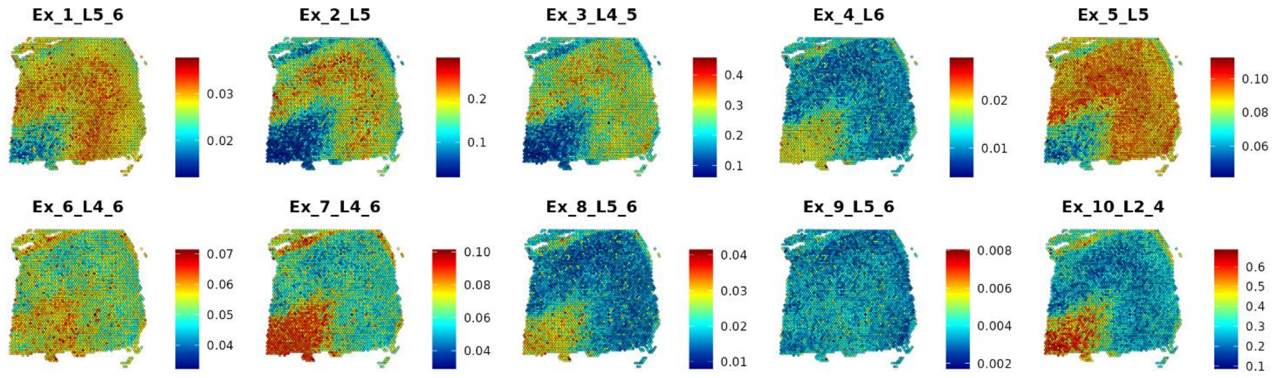

E **RCTD with the subpopulation**

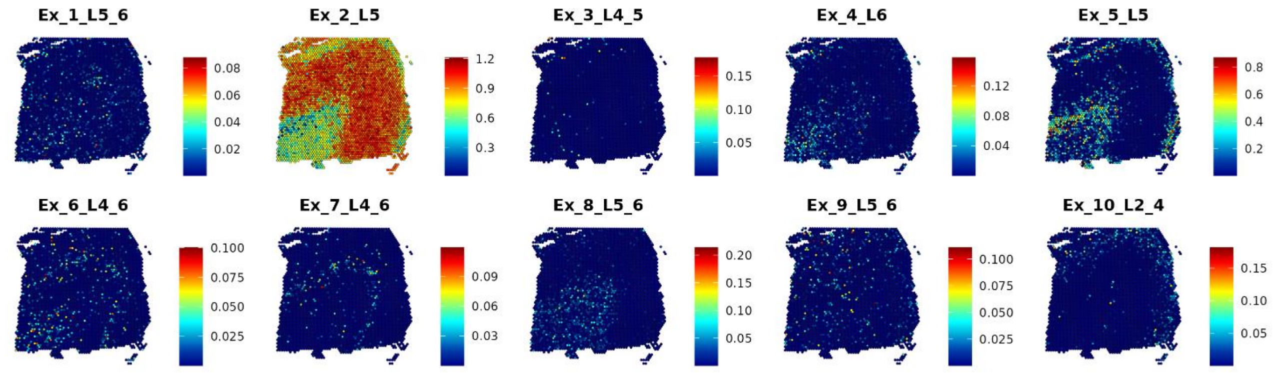

F **SPOTlight with the subpopulation**

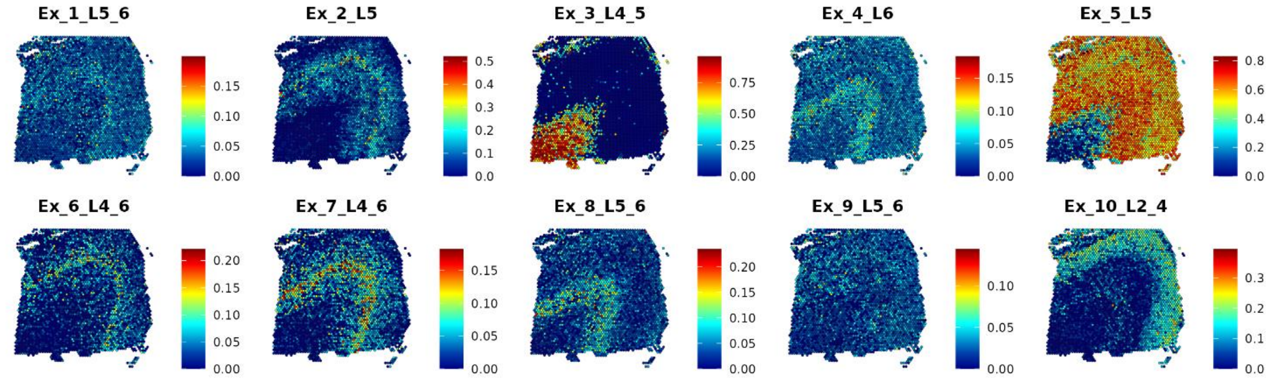

Fig. S5. Spatial distribution patterns of layer-specific neurons estimated by existing cell type deconvolution tools

The spatial composition of 10 layer-specific neuron types in human DLPFC tissue (slide number: 151673) was predicted by integrating spatial data with simulated subpopulation single-cell data. It was to assess the performance of the existing models, CARD, CellDART, Cell2location, DSTG, RCTD, and SPOTlight, in spatially mapping cell subpopulations. **(A)** In CARD, several cell types (Ex_2_L5, Ex_4_L6, Ex_7_L4_6, and Ex_8_L5_6) were highly distributed to the expected cortical layers while many of the cell types showed lower cell fraction in the corresponding layer than in other layers. **(B)** In CellDART, the neuron types were localized to a corresponding cortical layer in the majority of the cases; however, it failed to reflect the layer-specificity in Ex_3_L4_5 and Ex_10_L2_4. **(C)** In Cell2location, the cell types were highly restricted to the expected layers except for Ex_1_L5_6, Ex_4_L6, Ex_5_L5, and Ex_9_L5_6. **(D)** In DSTG, the cell types were restricted to the expected layers in Ex_1_5_6, Ex_2_L5, Ex_3_L4_5, and Ex_5_L5 while other cell types showed nonspecific distribution patterns. **(E)** In RCTD, Ex_2_L5 and Ex_10_L2_4 presented localized patterns of distribution in the corresponding layers; however, most of the other cell types had noisy or nonspecific spatial patterns. **(F)** In SPOTlight, the majority of neuron types revealed spatially restricted patterns while some cell types including Ex_3_L4_5 and Ex_9_L5_6 had lower cell fractions in the expected layers compared to other locations.

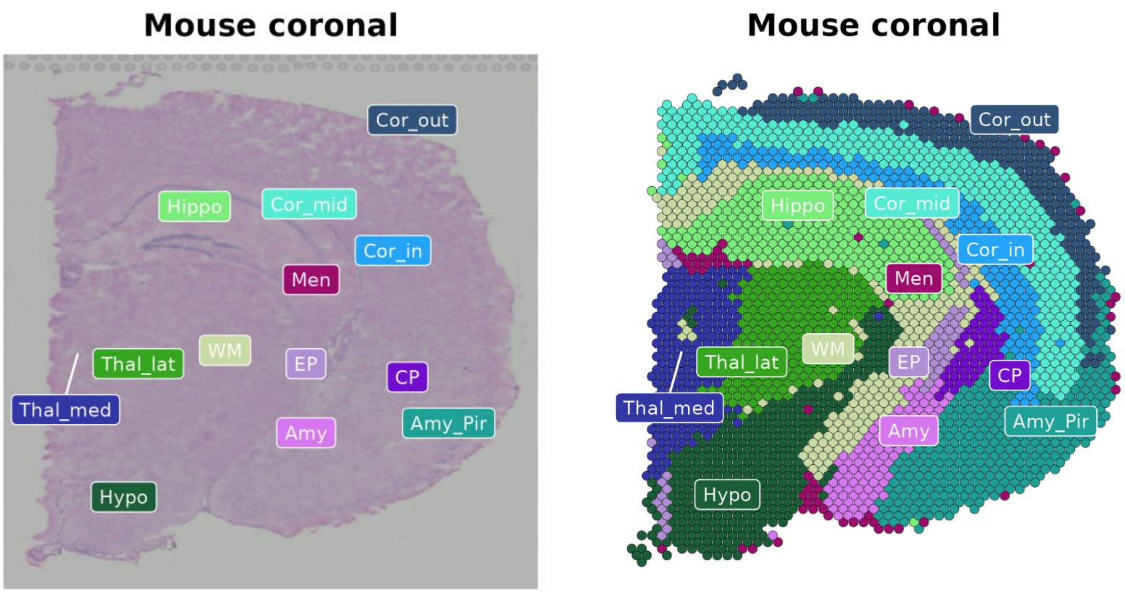

Fig. S6. Spot clustering of mouse brain spatial transcriptome

The spots comprising the spatial data were clustered based on the expression of highly variable genes (HVGs). Then, the resulting clusters were renamed according to the anatomical locations and visualized on top of the tissue. Amy: amygdala, Amy_Pir: amygdala or piriform cortex, Cor_out: outer cortex, Cor_mid: mid cortex, Cor_in: inner cortex, CP: caudoputamen, EP: ependyma, Hippo: hippocampus, Hypo: hypothalamus, Men: meninges, Thal_lat: lateral thalamus, Thal_med: medial thalamus, and WM: white matter.

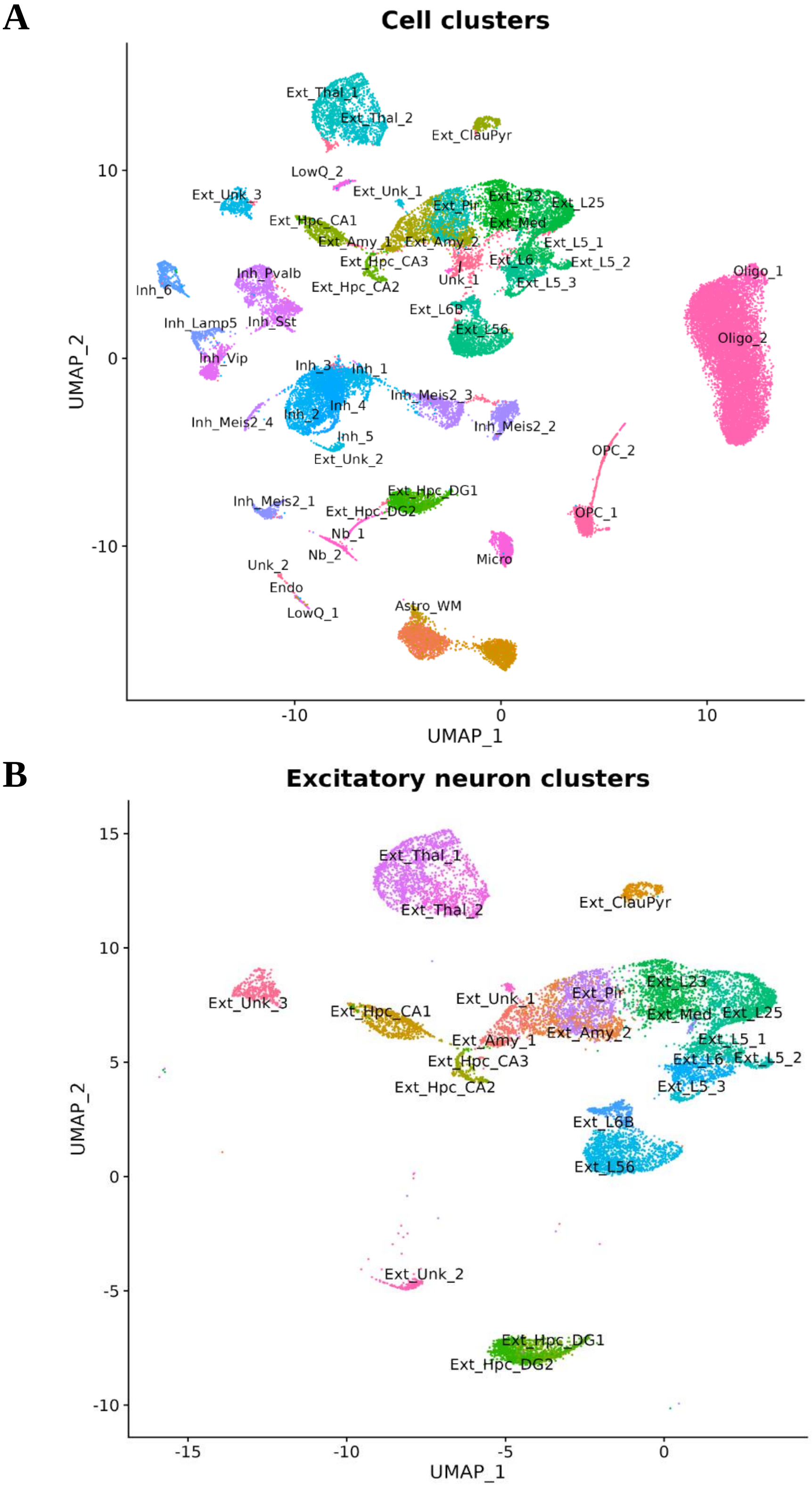
A
Cell clusters
Ext_Thal_1
Ext_Thal_2
Ext_ClauPyr
LowQ_2
Ext_Unk_1
Ext_Unk_3
Ext_Hpc_CA1
Ext_Pir
Ext_L23
Ext_L25
Ext_Med
Ext_Amy_1
Ext_Amy_2
Ext_L5_1
Ext_Hpc_CA3
Ext_L6
Ext_L5_2
Oligo_1
Unk_1
Ext_L5_3
Inh_Pvalb
Ext_Hpc_CA2
Inh_6
Ext_L6B
Inh_Lamp5
Inh_Sst
Ext_L56
Oligo_2
Inh_Vip
Inh_3
Inh_1
Inh_Meis2_3
Inh_4
Inh_2
Inh_Meis2_4
Inh_Meis2_2
Inh_5
OPC_2
Ext_Unk_2
Ext_Hpc_DG1
Inh_Meis2_1
Ext_Hpc_DG2
OPC_1
Nb_1
Nb_2
Micro
Unk_2
Endo
LowQ_1
Astro_WM
10
0
-10
-10
0
10
UMAP_2
UMAP_1
B
Excitatory neuron clusters
Ext_Thal_1
Ext_Thal_2
Ext_ClauPyr
Ext_Unk_3
Ext_Hpc_CA1
Ext_Unk_1
Ext_Pir
Ext_L23
Ext_Med
Ext_L25
Ext_Amy_1
Ext_Amy_2
Ext_L5_1
Ext_Hpc_CA3
Ext_L6
Ext_L5_2
Ext_Hpc_CA2
Ext_L5_3
Ext_L6B
Ext_L56
Ext_Unk_2
Ext_Hpc_DG1
Ext_Hpc_DG2
15
10
5
0
-5
-10
-15
-10
-5
0
UMAP_2
UMAP_1

Fig. S7. Single-nucleus data for the mouse brain coronal section

(A) Dimensionality reduction was performed and the cells composing single-nucleus data were visualized on a UMAP plot. The cell type annotation was color-coded on the plot.

(B) The 23 region-specific excitatory neurons were selected from a total of 59 cell types in the brain. The opted neuron types were visualized on UMAP and were utilized as a simulation dataset for the sorted single-cell data.

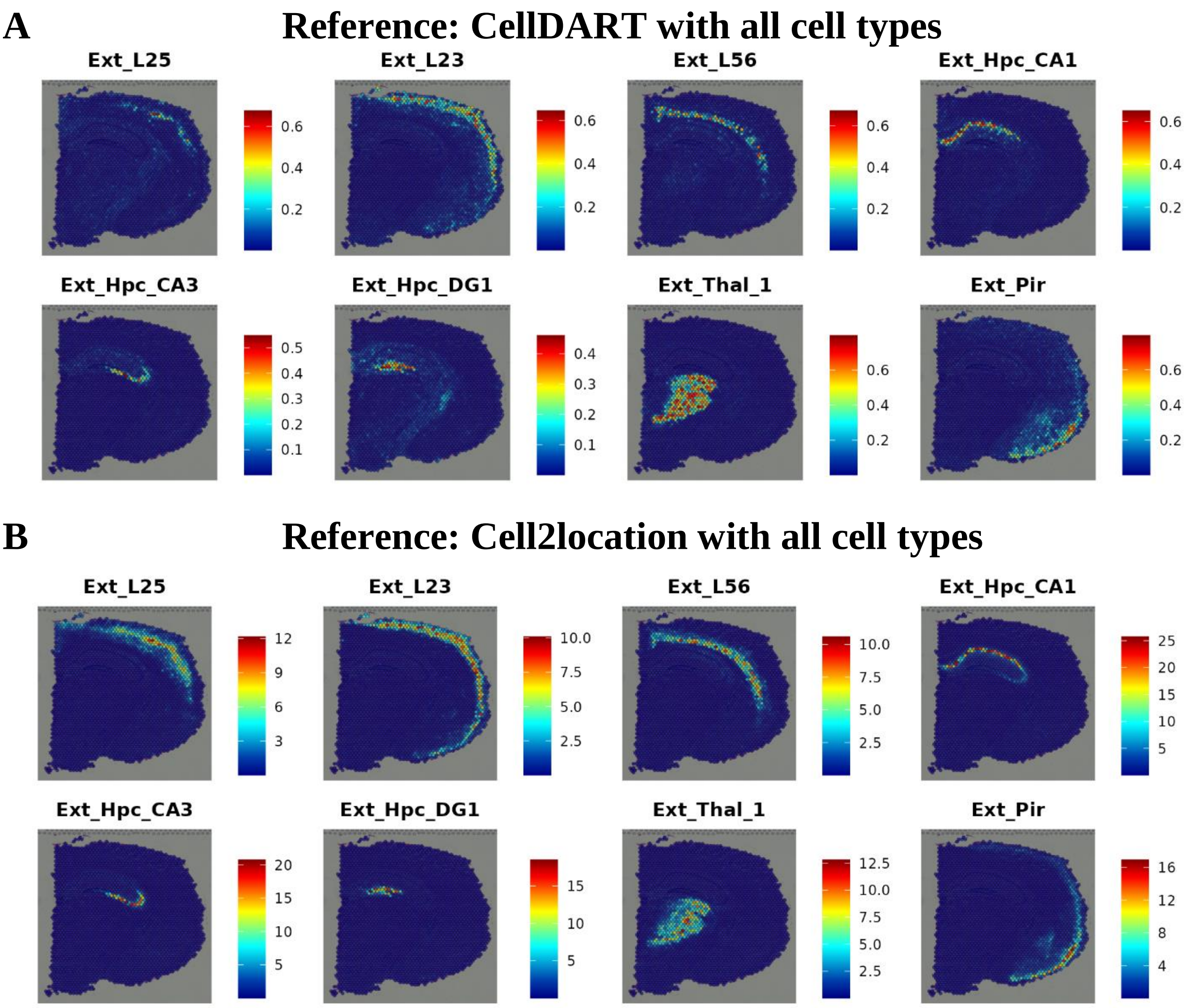

Fig. S8. Spatial maps of region-specific neurons in mouse brain using original single-cell data containing all cell types: the representative neuron types

The representative excitatory neuron types were spatially mapped to the tissue using original single-cell data containing all cell types. The spatial maps from the two methods, CellDART and Cell2location, were considered references to evaluate the prediction results of spSeudoMap. For both **(A)** CellDART and **(B)** Cell2location, the neuron types were spatially localized to the expected anatomical regions: Ext_L25, Ext_L23, and Ext_L56 to corresponding cortical layers, Ext_Hpc_CA1, Ext_Hpc_CA3, and Ext_Hpc_DG1 to the hippocampus, Ext_Thal_1 to the thalamus, and Ext_Pir to piriform cortex.

A **Reference: CellDART with all cell types**

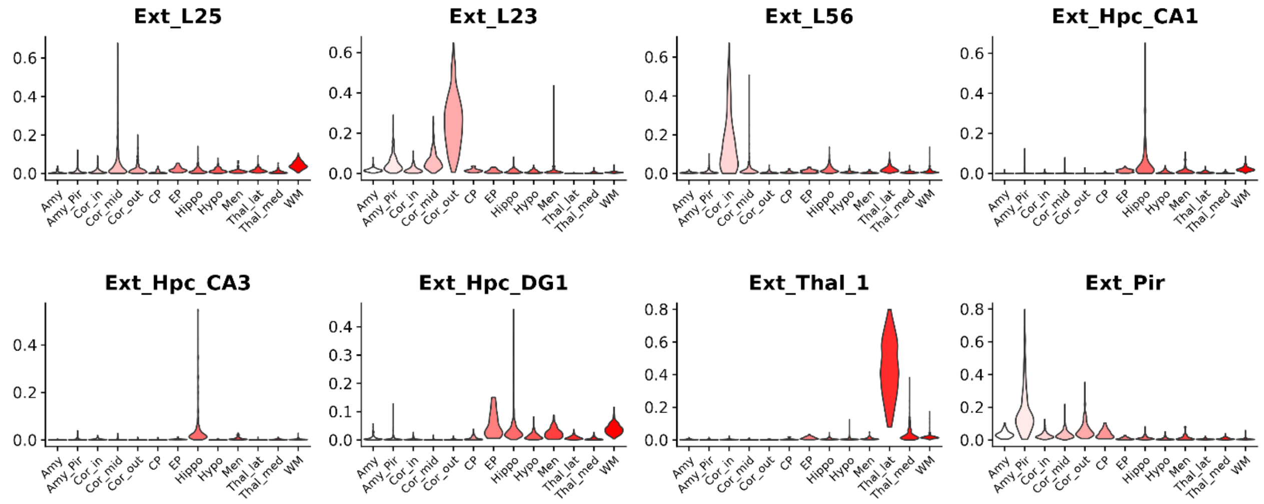

B **Reference: Cell2location with all cell types**

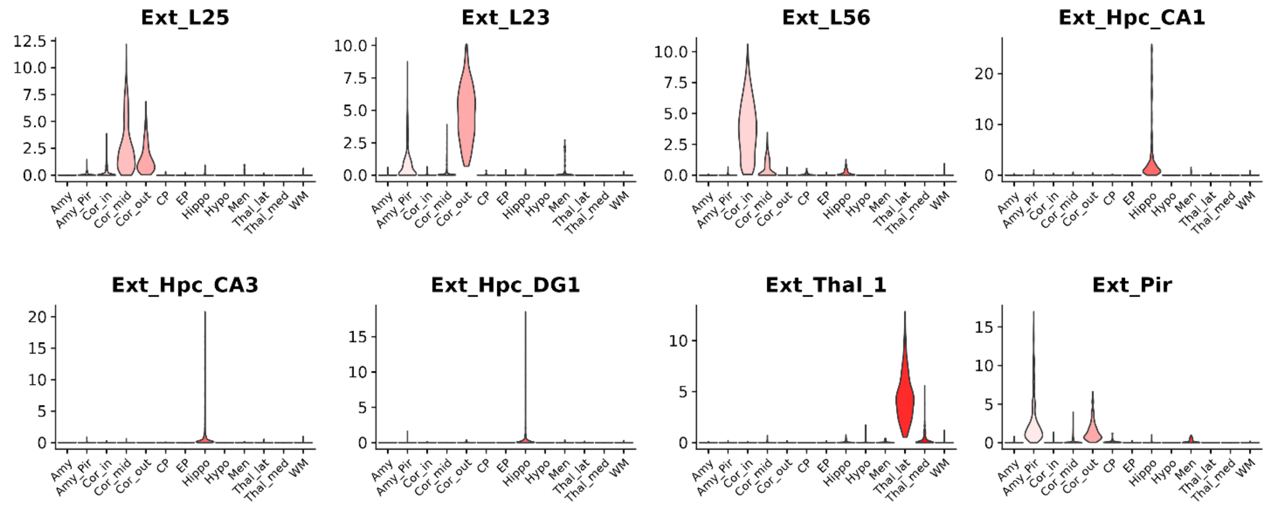

Fig. S9. Distribution of the neuron subtypes in mouse brain across the locations predicted from single-cell data covering all cell types

The region-specific excitatory neuron types were mapped to the tissue by CellDART and Cell2location. Violin plots represent the predicted excitatory neuron fraction in the corresponding spot clusters. In both **(A)** CellDART and **(B)** Cell2location, the cell subtypes were accurately localized to the expected anatomical locations. Amy: amygdala, Amy_Pir: amygdala or piriform cortex, Cor_out: outer cortex, Cor_mid: mid cortex, Cor_in: inner cortex, CP: caudoputamen, EP: ependyma, Hippo: hippocampus, Hypo: hypothalamus, Men: meninges, Thal_lat: lateral thalamus, Thal_med: medial thalamus, and WM: white matter.

A Reference: CellDART with all cell types

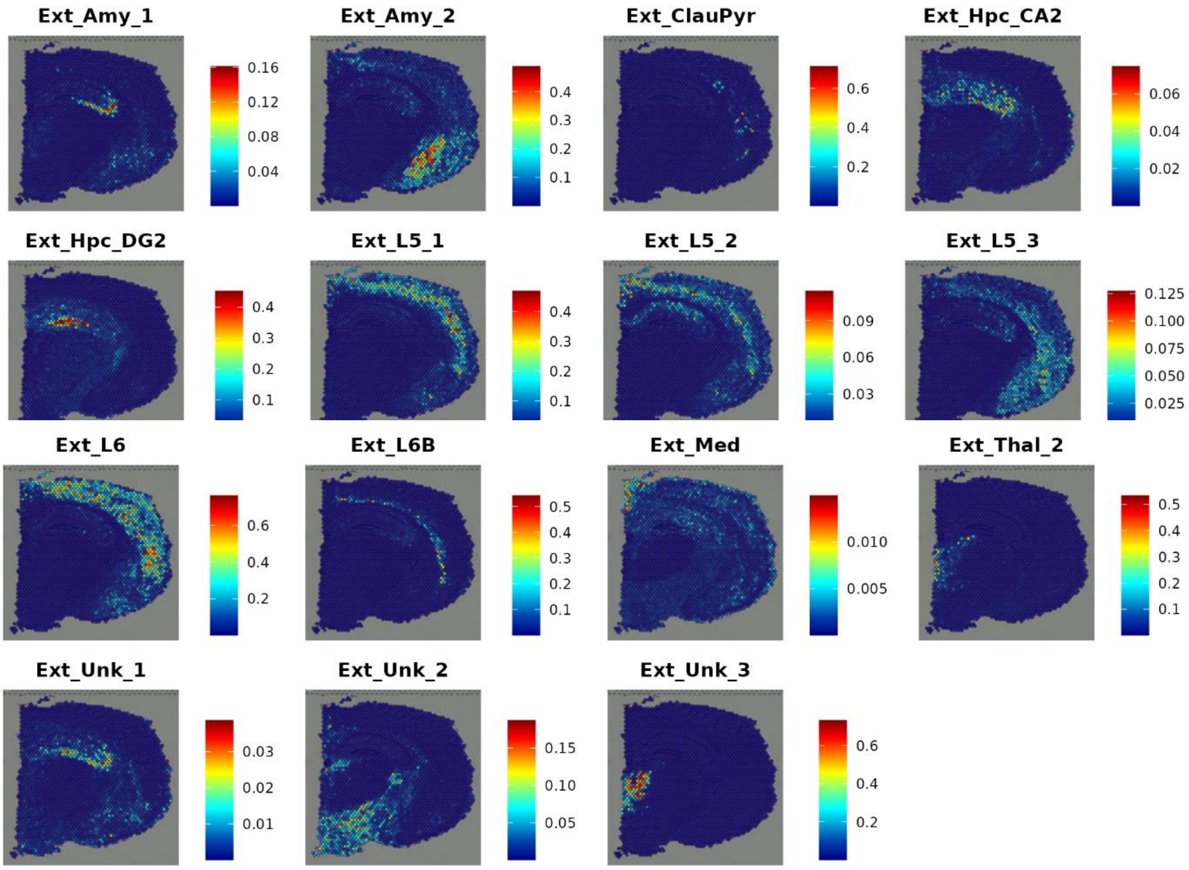

B **Reference: Cell2location with all cell types**

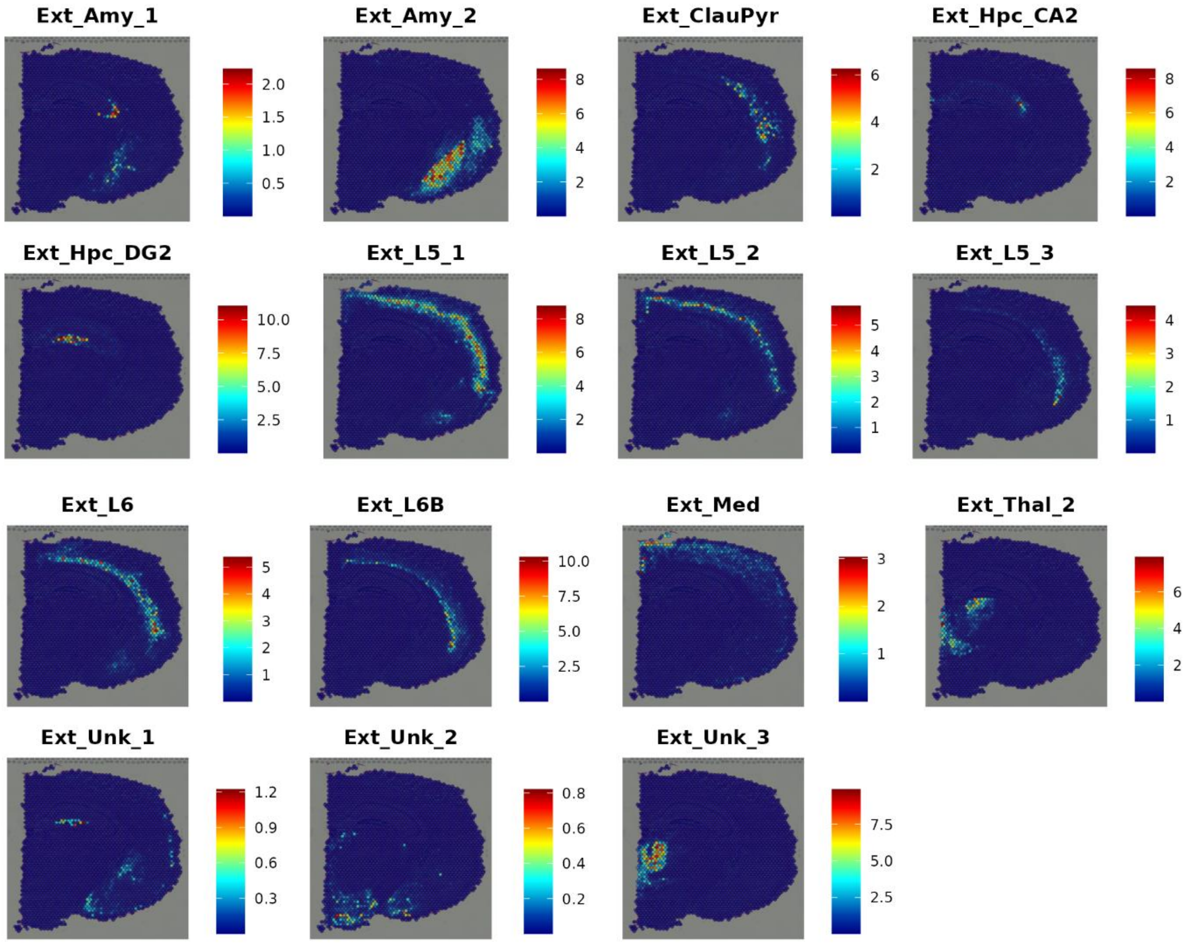

C spSeudoMap with the subpopulation

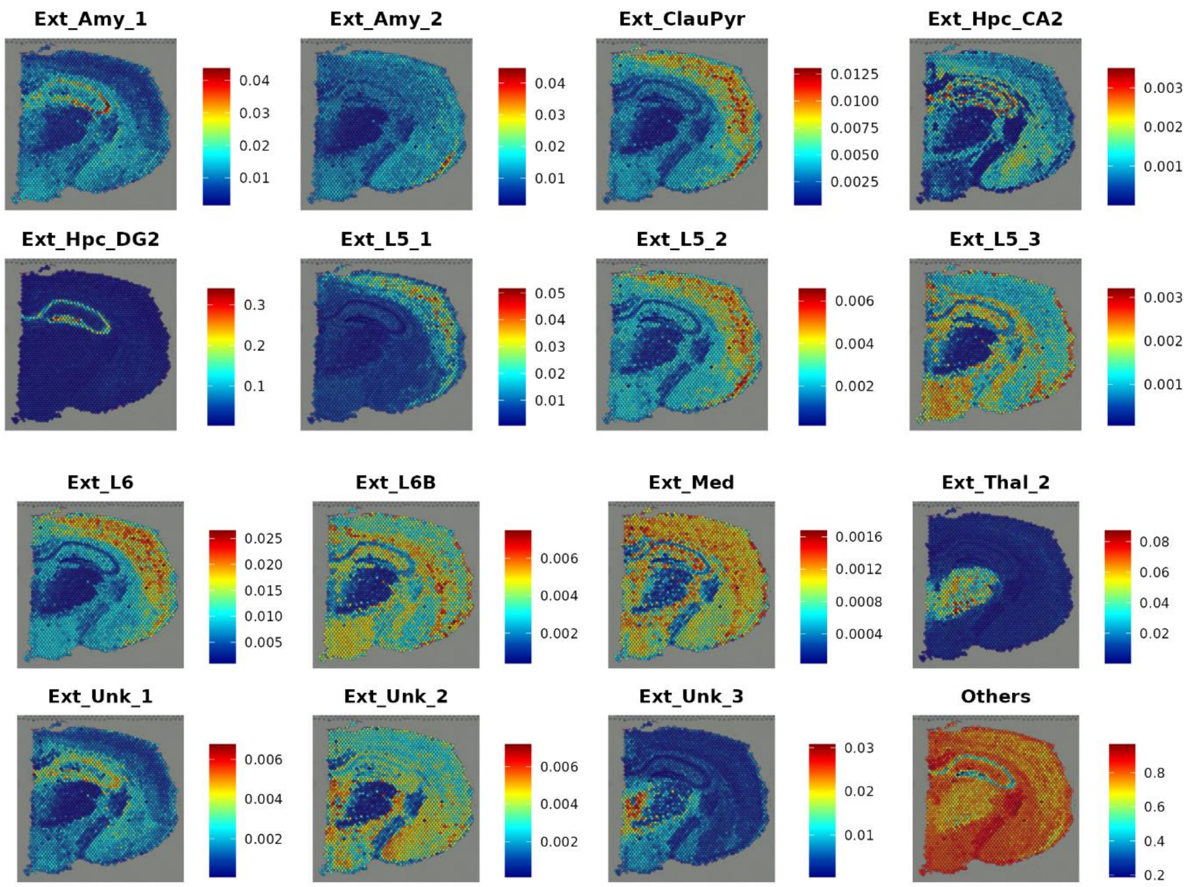

Fig. S10. The spatial landscape of region-specific neurons in mouse brain: rest of the neuron types

The spatial distribution of the rest of the region-specific neuron types in the mouse brain was predicted by integrating spatial data with single-cell data covering all cell types using **(A)** CellDART and **(B)** Cell2location. The spatial maps created by both tools were considered references and were compared with the cell subpopulation mapping results from **(C)** spSeudoMap. In both **(A)** CellDART and **(B)** Cell2location, the layer-specific neurons were localized to the corresponding cortical layers, hippocampal neurons to the hippocampus, and thalamic neurons to the thalamus (except for Ext_Unk neuron types whose regional specificity is not known). Overall the two reference methods showed similar patterns of spatial distribution. **(C)** In the case of spSeudoMap, the rest of the neuron types showed high cellular fractions in the corresponding anatomical locations (except for Ext_Unk neuron types whose regional specificity is not known). However, the proportion was also high in nonspecific regions, particularly in the cell types with low cell fractions.

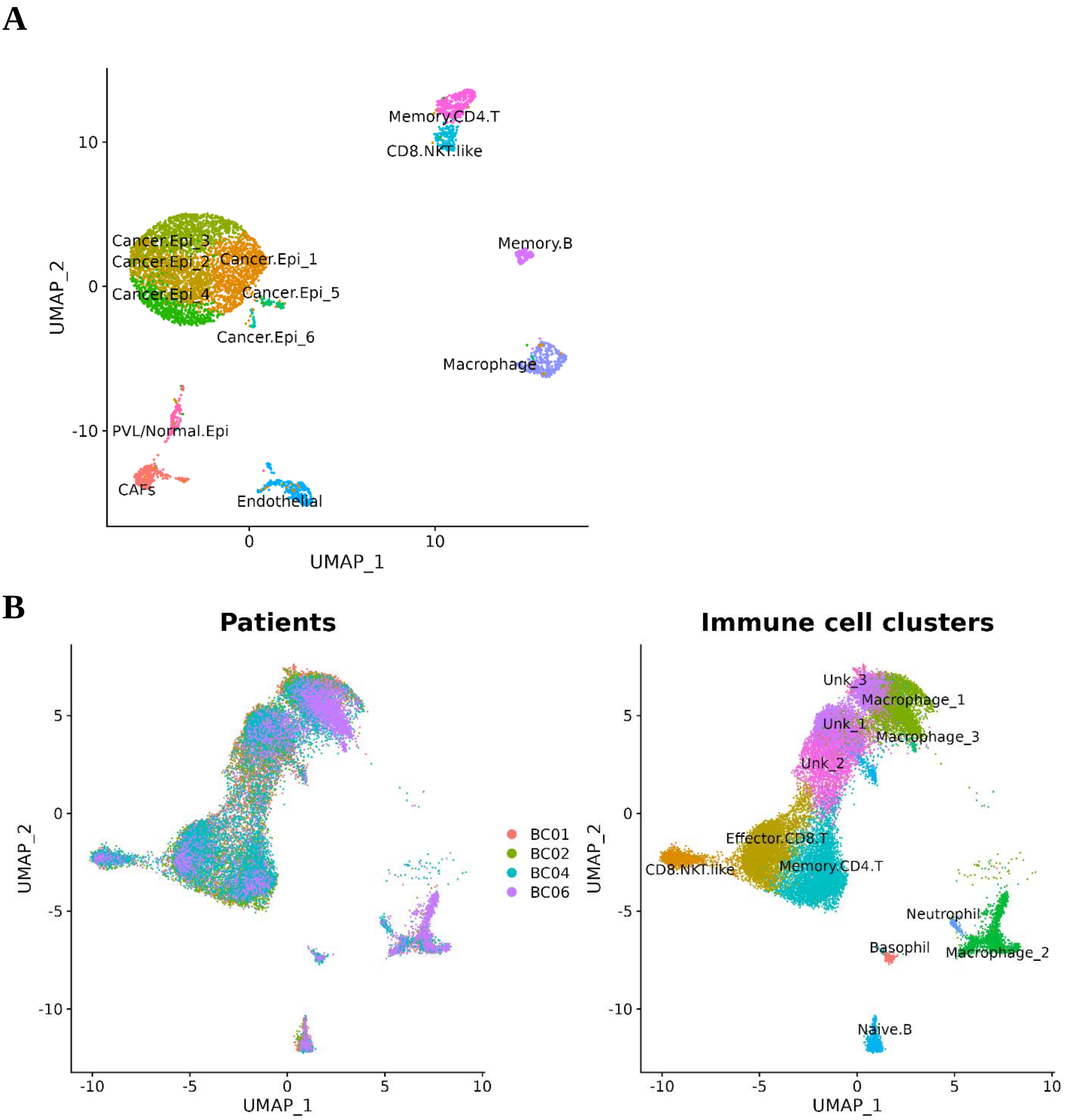
A
Memory.CD4.T
CD8.NKT.like
Memory.B
Cancer.Epi_3
Cancer.Epi_2
Cancer.Epi_1
Cancer.Epi_4
Cancer.Epi_5
Cancer.Epi_6
Macrophage
PVL/Normal.Epi
CAFs
Endothelial
UMAP_1
UMAP_2
B
Patients
BC01
BC02
BC04
BC06
UMAP_1
UMAP_2
Immune cell clusters
Unk_3
Macrophage_1
Unk_1
Macrophage_3
Unk_2
Effector.CD8.T
CD8.NKT.like
Memory.CD4.T
Neutrophil
Basophil
Macrophage_2
Naive.B
UMAP_1
UMAP_2

Fig. S11. Single-cell data for the human breast cancer

(A) Dimensionality reduction was performed for the matched single-cell data of the same patient. The gene expression profile of cells was visualized with a UMAP plot and the cell types were color-coded. The dataset was considered as a reference for the evaluation of spatial cell composition.

(B) Dimensionality reduction was done and the single-cell data obtained by CD45+ sorting was exhibited using a UMAP plot. Only the 12 immune cell types were selected and visualized. Then the immune cell types were spatially mapped to the tissue using spSeudoMap.

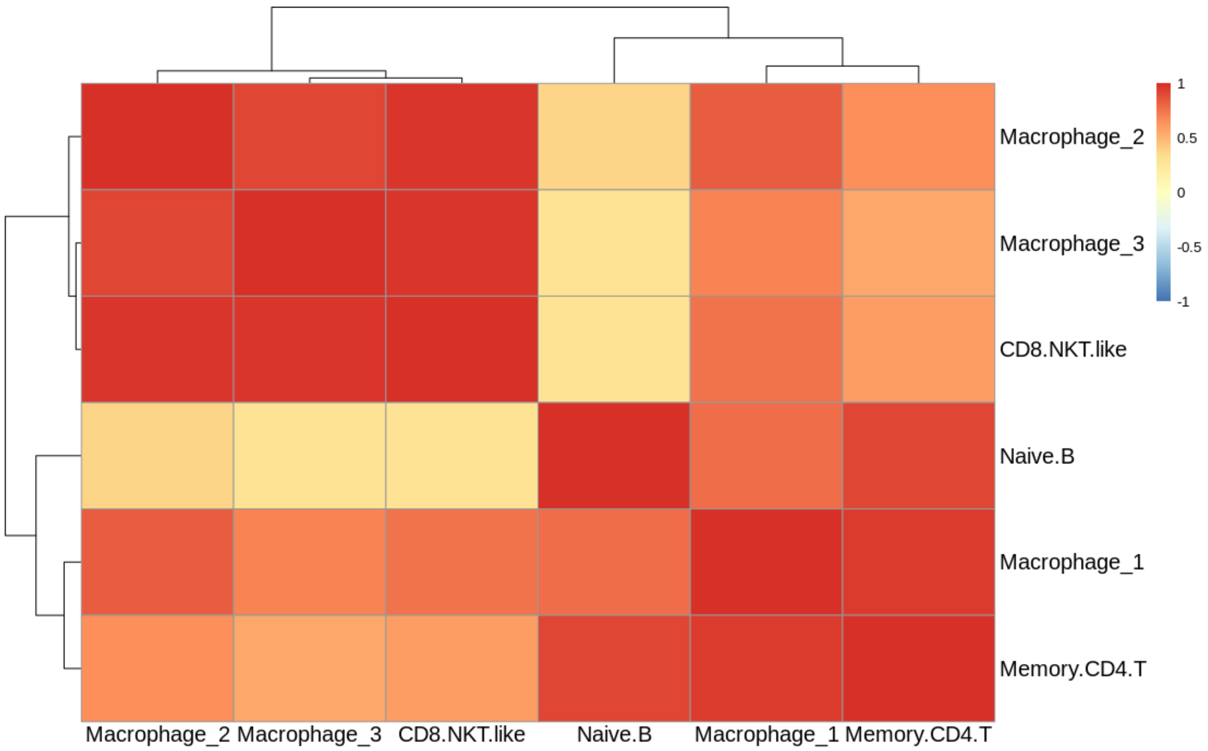

Fig. S12. Spatial correlation patterns between immune cells in the human breast cancer tissue

Spearman's correlation coefficients were calculated between the predicted cell fraction of the top 8 cell types. The proximity of the spatial correlation patterns was examined and hierarchical clustering was performed. Memory CD4 T cells and macrophage_1, and CD8+ NKT-like cells and macrophage_3 showed high similarities among the cell type pairs.

A

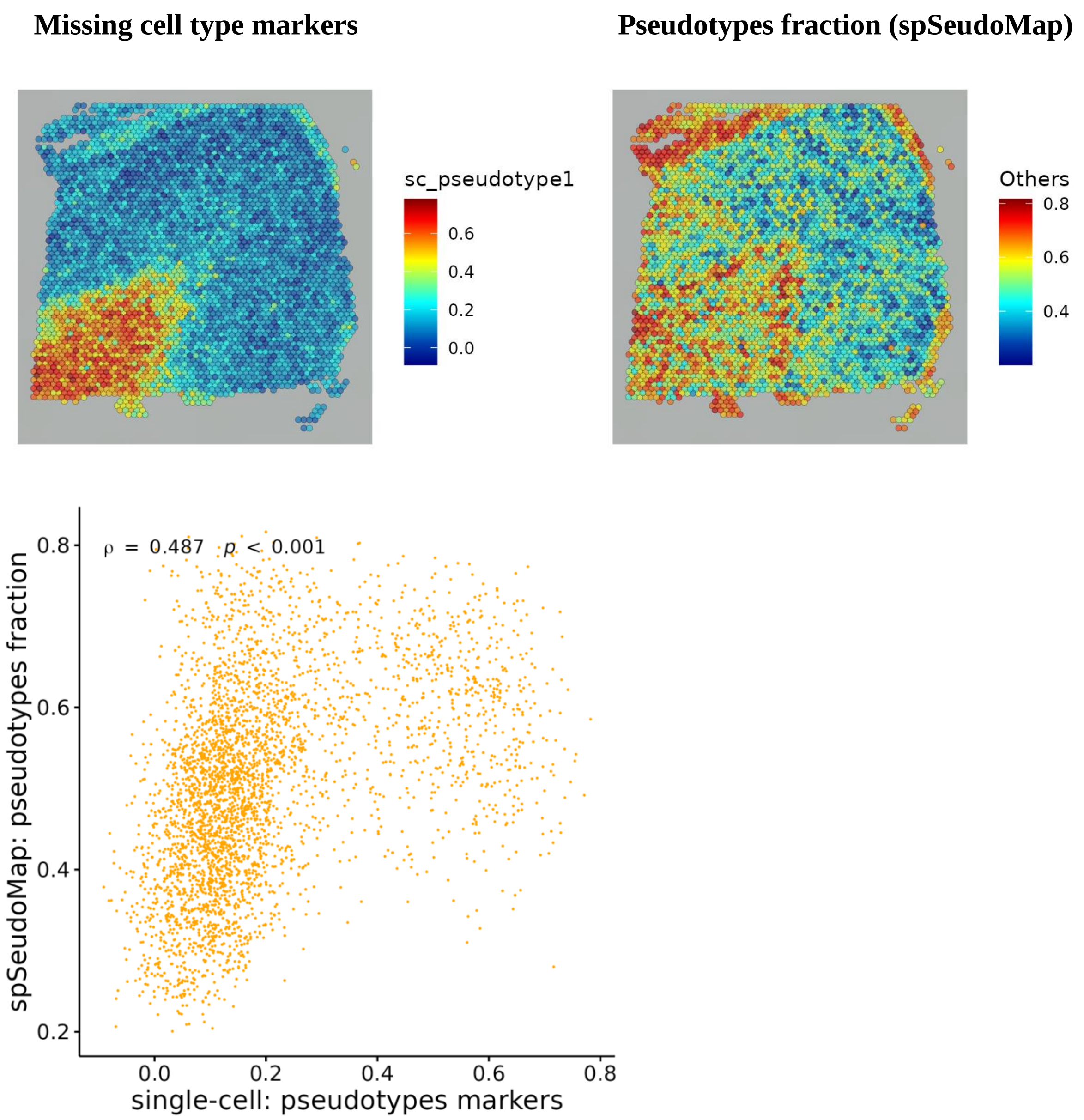

Missing cell type markers
Pseudotypes fraction (spSeudoMap)
sc_pseudotype1
0.6
0.4
0.2
0.0
Others
0.8
0.6
0.4
ρ = 0.487 p < 0.001
spSeudoMap: pseudotypes fraction
single-cell: pseudotypes markers
0.8
0.6
0.4
0.2
0.0
0.2
0.4
0.6
0.8

B

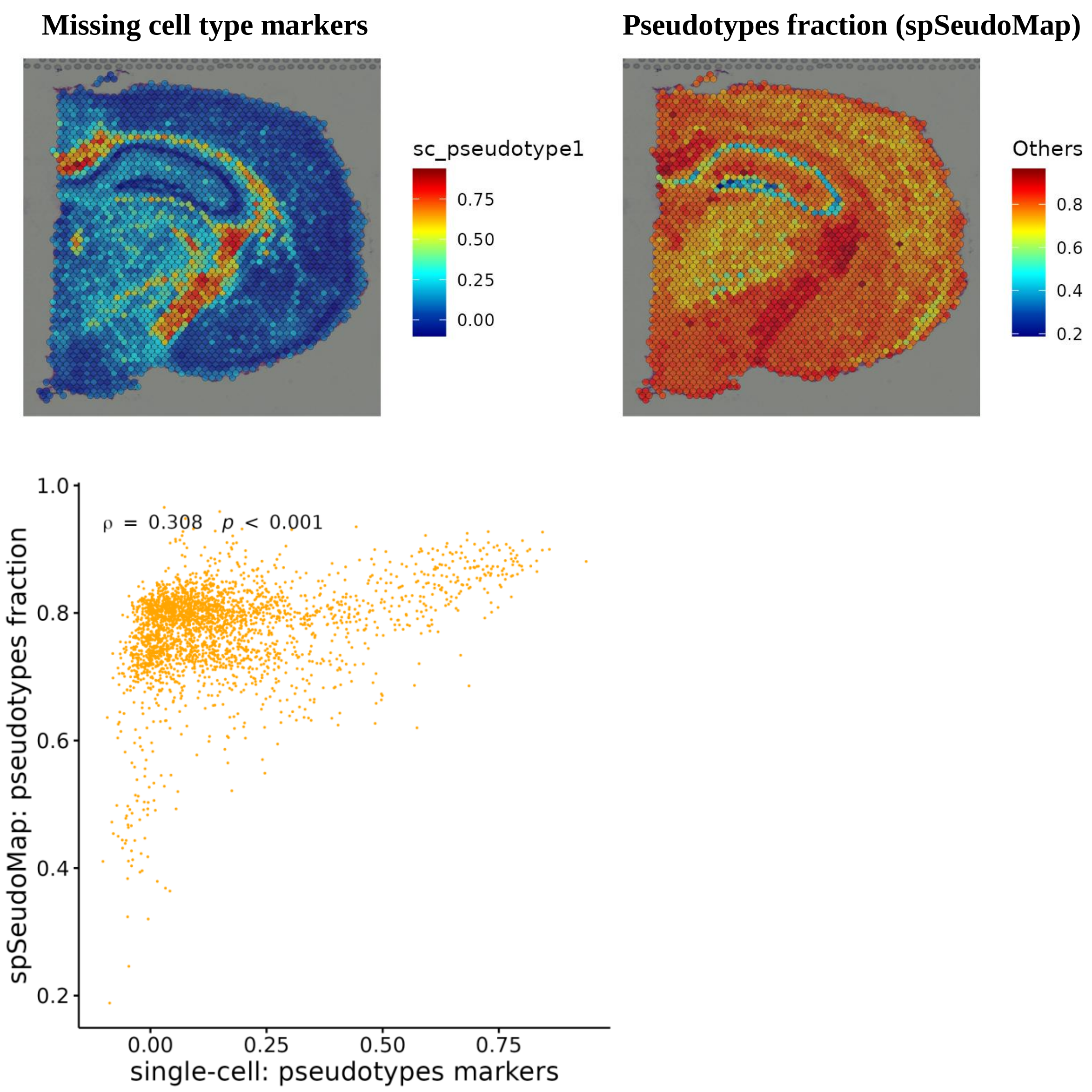

Missing cell type markers
sc_pseudotype1
0.75
0.50
0.25
0.00
Pseudotypes fraction (spSeudoMap)
Others
0.8
0.6
0.4
0.2
ρ = 0.308 p < 0.001
1.0
0.8
0.6
0.4
0.2
spSeudoMap: pseudotypes fraction
0.00
0.25
0.50
0.75
single-cell: pseudotypes markers

Fig. S13. Comparison between spatial enrichment patterns for marker genes of missing cell types in single-cell data and the pseudotypes fraction predicted from spSeudoMap.

The two different measures which explain the spatial distribution of missing cell types in single-cell data were compared in brain tissues: enrichment score of missing cell types markers and pseudotype fraction predicted by spSeudoMap. In the top-left panel and the x-axis of the scatter plots, the enrichment scores calculated from the differentially expressed genes between excitatory neurons and other populations were presented. In the top-right panel and the y-axis of the scatter plots, pseudotypes fraction predicted as the output of spSeudoMap was exhibited. Spearman's correlation coefficients and statistical significance were calculated and presented in the top-left corner of each plot. The two fraction values showed positive correlations in **(A)** the human brain (slide number: 151673) and **(B)** mouse brain tissues.

A

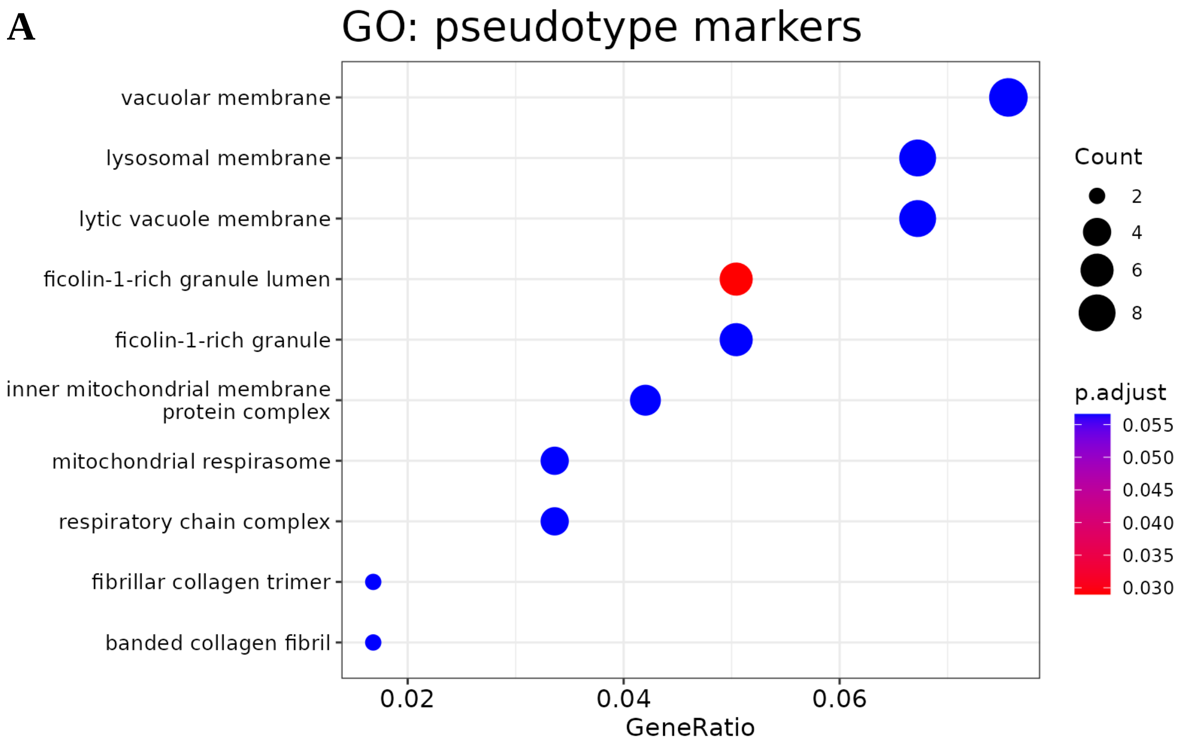

GO: pseudotype markers
vacuolar membrane
lysosomal membrane
lytic vacuole membrane
ficolin-1-rich granule lumen
ficolin-1-rich granule
inner mitochondrial membrane protein complex
mitochondrial respirasome
respiratory chain complex
fibrillar collagen trimer
banded collagen fibril
0.02
0.04
0.06
GeneRatio
Count
2
4
6
8
p.adjust
0.055
0.050
0.045
0.040
0.035
0.030

B

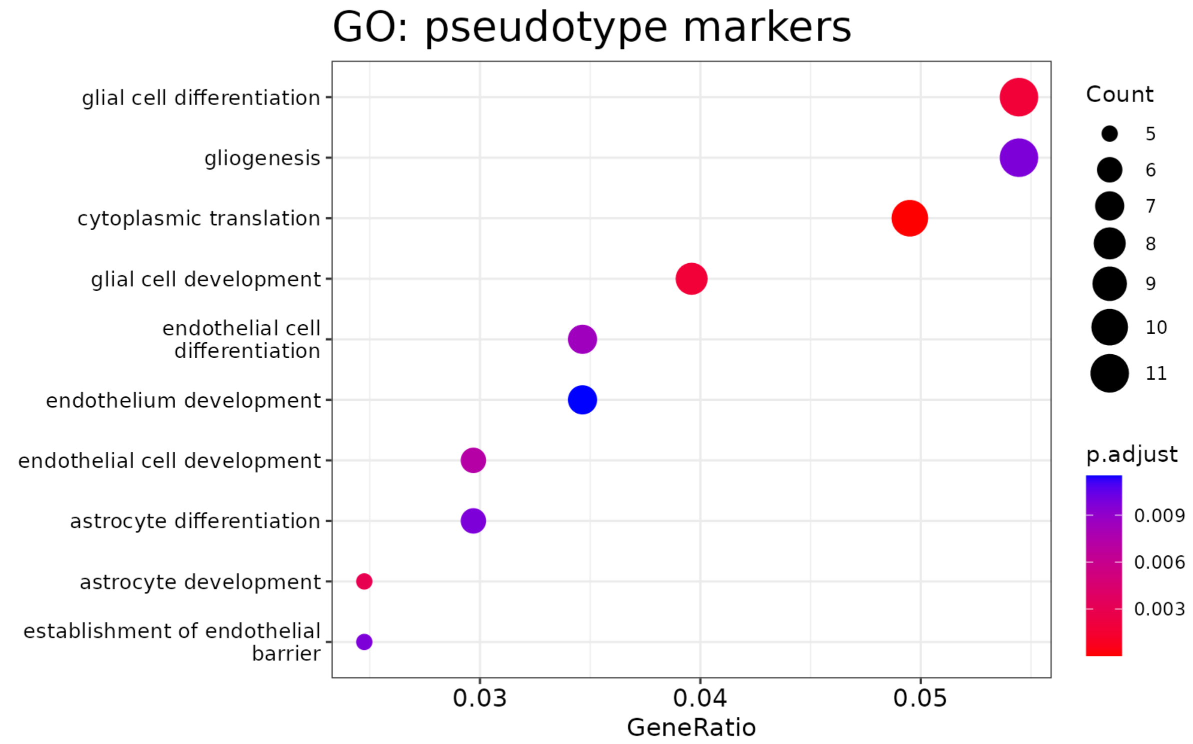

GO: pseudotype markers
glial cell differentiation
gliogenesis
cytoplasmic translation
glial cell development
endothelial cell differentiation
endothelium development
endothelial cell development
astrocyte differentiation
astrocyte development
establishment of endothelial barrier
0.03
0.04
0.05
GeneRatio
Count
5
6
7
8
9
10
11
p.adjust
0.009
0.006
0.003

C

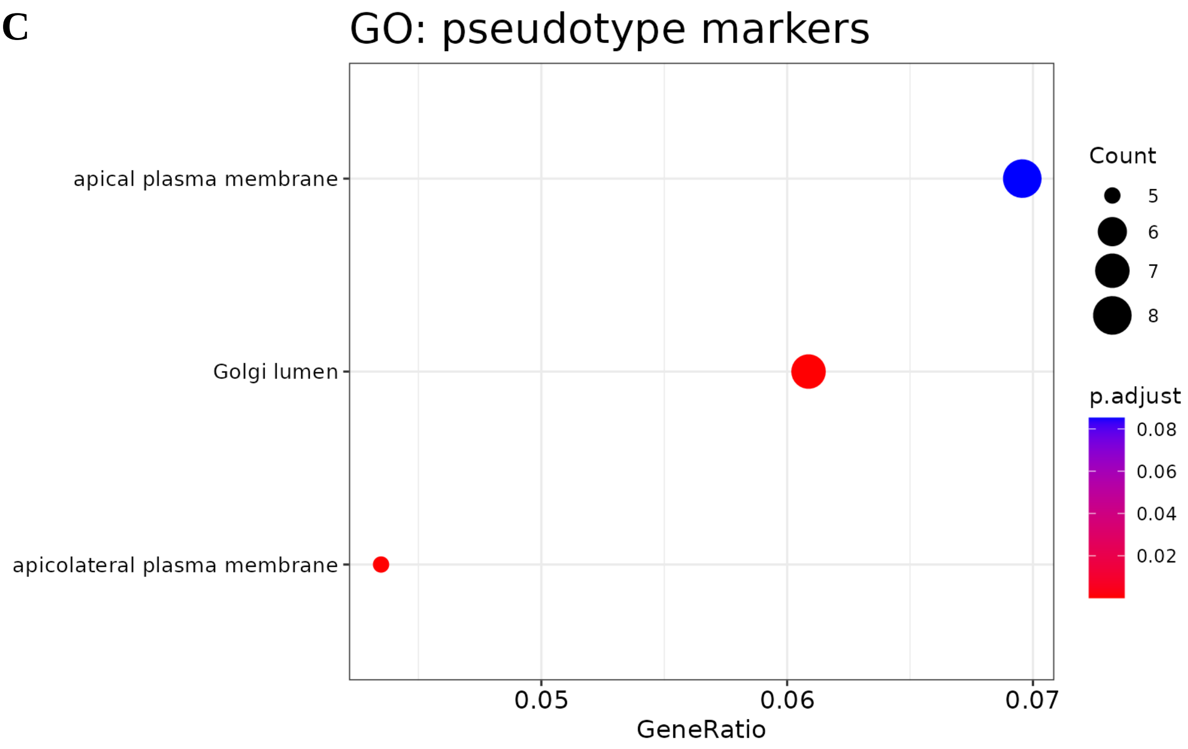

Fig. S14. Functional implication of pseudotype markers extracted from spSeudoMap

The over-representation analysis was performed with R library clusterProfiler to discover the Gene Ontology (GO) terms associated with pseudotype markers extracted by spSeudoMap [1-3]. The results were visualized with a dotplot and the top 10 GO terms having the highest gene ratio were presented. Gene counts give the number of overlapping genes between pseudotype markers and each GO term, and gene ratio is the ratio of the gene counts to the number of markers. For multiple comparison correction, the false discovery rate (FDR) was controlled below 0.1 based on the Benjamini-Hochberg method. **(A)** In the human brain, **(B)** mouse brain, and **(C)** human breast cancer tissues, the pseudotype markers were not related to cell proliferation or cell metabolism.

References

1. Gene Ontology C. The Gene Ontology resource: enriching a GOld mine. Nucleic Acids Res. 2021;49:D325-D334.
2. Ashburner M, Ball CA, Blake JA, Botstein D, Butler H, Cherry JM, et al. Gene ontology: tool for the unification of biology. The Gene Ontology Consortium. Nat Genet. 2000;25:25-29.
3. Wu T, Hu E, Xu S, Chen M, Guo P, Dai Z, et al. clusterProfiler 4.0: A universal enrichment tool for interpreting omics data. Innovation (Camb). 2021;2:100141.
